# Deep Learning for Root Canal Filling Quality on Panoramic Radiographs with Detection and Grading Measured Separately

**DOI:** 10.3390/diagnostics16203371

**Published:** 2026-10-16

**Authors:** Faruk Oztekin, Oguzhan Katar, Mehmet Eskibaglar, Furkan Konus, Mehmet Rasit Atik, Nurefsan Ozdogan, Sumeyye Cosgun Baybars

**Affiliations:** 1 Department of Endodontics, Faculty of Dentistry, Firat University, Elazig 23119, Turkey; meskibaglar@firat.edu.tr (M.E.); mratik@firat.edu.tr (M.R.A.); navci@firat.edu.tr (N.O.); sbaybars@firat.edu.tr (S.C.B.); 2 Department of Software Engineering, Firat University, Elazig 23119, Turkey; okatar@firat.edu.tr; 3 Department of Endodontics, Faculty of Dentistry, Yozgat Bozok University, Yozgat 66100, Turkey; furkan.konus@bozok.edu.tr

**Keywords:** endodontics, treatment outcome assessment, object detection, convolutional neural network, label noise, explainable artificial intelligence, computer-aided diagnosis, dental informatics

## Abstract

**Background/Objectives:** The European Society of Endodontology names two filling requirements with a reproducible link to outcome: apical extent and material density. Panoramic radiography shows every treated tooth in one exposure, but systems built on it pool detection with grading into one average precision that hides which of the two has failed. This study graded both axes on panoramic radiographs with detection and discrimination measured separately. **Methods:** In this retrospective study, two specialist endodontists read 830 panoramic radiographs independently, and a third adjudicated every disagreement. The 1668 root fillings of 806 radiographs were encoded into a binary and an axis-resolved label space, and 160 radiographs were locked away before training. The remaining 646 were evaluated under five-fold cross-validation, with four architectural interventions tested alongside input resolution. Localisation was measured by class-agnostic recall, grading by matched-box accuracy and per-axis F1, and label spaces were compared by paired bootstrap. **Results:** Refining the label space left detection recall at 92.6% against 93.1%, but cost 11.41 points of matched-box accuracy, 8.20 to 14.46, and 12.16 on the locked test. Asked the same binary question, it fell 1.12 points behind in cross-validation and 3.13 points behind on the locked test, where the binary decision reached 76.25% sensitivity given detection and 68.75% specificity at the best of ten readings, or 71.52% and 68.21% at the development-selected one. At 1024 pixels, no architectural change improved density F1 beyond the 8.2 to 10.8 points this design can detect, and enlarging training from 207 to 517 radiographs gained 11.42, 5.92 to 16.56. **Conclusions:** Separating detection from grading locates the cost of a finer label space in discrimination, bounds four architectural interventions below the resolution of this design, and supports a binary but not an axis-resolved quality decision as a potential teaching and audit aid, pending external validation beyond this single centre, not a screening test.

## 1. Introduction

A root canal filling occupies the prepared canal space once the pulp has been removed. Its job is to seal that space against reinfection, whether from the oral cavity or from the periradicular tissues. Because that seal cannot be inspected directly, its technical quality has to be judged on radiographs against a small set of agreed criteria. The European Society of Endodontology states these as a narrative list rather than as a graded scheme. Among them, the canal should be filled completely, with no space between the filling and the canal wall, and the working length to which it is filled should lie 0.5 to 2 mm short of the radiographic apex [1]. Two of those requirements carry a reproducible association with outcome: the apical extent of the filling and the density of the material. Radiographic filling quality is itself only one component of treatment quality, since the radiograph shows the filling and not how the canal was accessed, shaped, and disinfected before it.

Each axis carries that association on its own. Fillings of inadequate apical extent have been linked to apical periodontitis across independent cross-sectional cohorts, with odds ratios of roughly 1.6 to 2.6 [2,3,4,5]. Non-homogeneous fillings likewise show a higher prevalence of apical periodontitis than homogeneous ones, 58.6% against 41.9% [2]. Neither deficiency is rare: a cross-sectional survey of 614 panoramic radiographs rated 54% of root-filled teeth inadequate [6].

Even at that prevalence, clinicians still assess these fillings one tooth at a time and by eye. This bounds how many radiographs a prevalence survey or a clinical audit can cover. It also admits the observer variability that has been reported repeatedly: when two examiners score the same images independently [7], when examiners are scored against an expert reference standard [8], and when several readers score related radiographic judgements on the same panoramic radiographs [9]. The same constraint operates in undergraduate education. There, student-performed fillings are documented on routine periapical radiographs and can be audited retrospectively in volume [10], and a panoramic reading triggers closer inspection on a periapical radiograph rather than a treatment decision. An automated reader would serve all three settings without any change to the imaging protocol, because a single routine exposure already carries every treated tooth in the arch.

Readers of that kind have already been built on the same projection for neighbouring tasks. Deep learning has addressed endodontically treated teeth [11,12], most often through the presence of a root filling, whereas periapical status has been classified by the same means on periapical radiographs [13] and periapical radiolucent lesions have been detected on panoramic radiographs [14]. A systematic review of thirteen studies covering 12,513 examinations reported sensitivity between 0.85 and 1.00 for detecting root canal fillings and between 0.79 and 1.00 for grading obturation quality. Performance in grading obturation quality was lower on panoramic radiographs than on periapical radiographs or cone beam computed tomography [15].

What these systems grade, however, is not the rubric a clinician applies. Work on filling quality has adopted flat schemes throughout. Some collapse every deficiency into a single class beside the adequate one [16,17], and the second of those scores apical extent only. Others put each criterion into a separate binary decision [18,19]. Where a multi-class scheme has been used at all, it covered the length axis alone [20]. Clinical judgement, by contrast, is staged: a filling is first acceptable or not, and only then deficient along a particular axis. No study has read one annotation at both of those levels, so what the second, axis-resolving level costs over the first has never been measured under a protocol that holds the rest of the pipeline fixed.

The metric these systems are reported by conceals the same distinction. Performance is usually summarised by average precision, in a literature where 42 different performance metrics have been counted across 168 studies, and a minimum core set has been called for [21]. Average precision aggregates localisation and classification, so a model that finds every root-filled tooth but assigns the wrong defect axis is penalised like one that finds no teeth at all. That aggregation suits this task particularly badly. A root filling is a dense radiopaque object against cancellous bone and close to trivial to localise, whereas the defect that determines its quality is a two-millimetre difference at the apical termination or a small radiolucent discontinuity inside the material. One number cannot say which of the two a method improved.

A third question survives both of these: which radiographic evidence such a model actually uses. Post hoc attribution is the usual instrument, but its standard class-discriminative form asks where the evidence for one class rather than another lies, and the bounding box has already answered that [22]. The question worth asking is whether the decision rests on the material inside the canal or on the coronal restoration above it. A void is a radiolucent discontinuity inside radiopaque filling material, and the black top-hat transform [23], gated by brightness, is used here to isolate that appearance. Attribution can therefore be scored against a physically defined target computed from the radiograph itself. Such a target is ordinarily assumed to mark the evidence it stands for, rather than shown to do so.

This study therefore takes up all three together, on one cohort of panoramic radiographs. The same annotations support both a binary adequate-versus-defective decision and an axis-resolved decision that separates a length axis from a density axis, so the two label spaces can be trained and read under an otherwise identical protocol. Localisation and discrimination are carried out separately rather than aggregated. Four architectural interventions are each referred to the smallest difference the design can detect, and the attribution reference is tested before anything is concluded from it.

The main contributions are as follows.

- The cost of resolving the defect axis is measured rather than assumed. Under an otherwise identical protocol, refining two nested classes into three leaves class-agnostic detection recall where it was, but gives up 11.41 points of matched-box classification accuracy. That cost replicates on a locked test set, and it traces to the axis assignment rather than to the quality decision beneath it.
- Localisation and discrimination are reported separately. Class-agnostic detection over every box, and discrimination over boxes already matched to the reference standard, are carried as separate quantities alongside the joint one. Under that separation, the ordering of two configurations reverses, against an ordering that macro-F1 holds at every threshold swept.
- Attribution is scored against an annotation-free reference derived from the physical definition of the criterion, and that reference is then tested in its own right. The attribution itself separates from an edge map and from a random map on the region-mass and pointing quantities. However, a model given the reference as an input channel attends to it no more than a model that was not, so a null against the reference is reported as a bounded non-detection and the reference is not treated as a validated marker of voids.
- The residual limit is placed by elimination rather than attributed to architecture. At 1024 pixels, four architectural interventions leave the density axis where they found it, over intervals that all contain zero and against a stated smallest detectable difference. Training set size, by contrast, moves that axis by more than the whole usable resolution range delivers.
- To the best of our knowledge, this is the first study to measure using panoramic radiography what resolving the defect axis costs over a binary quality decision read from the same annotation, with localisation and discrimination reported separately.

## 2. Materials and Methods

**Figure 1:**
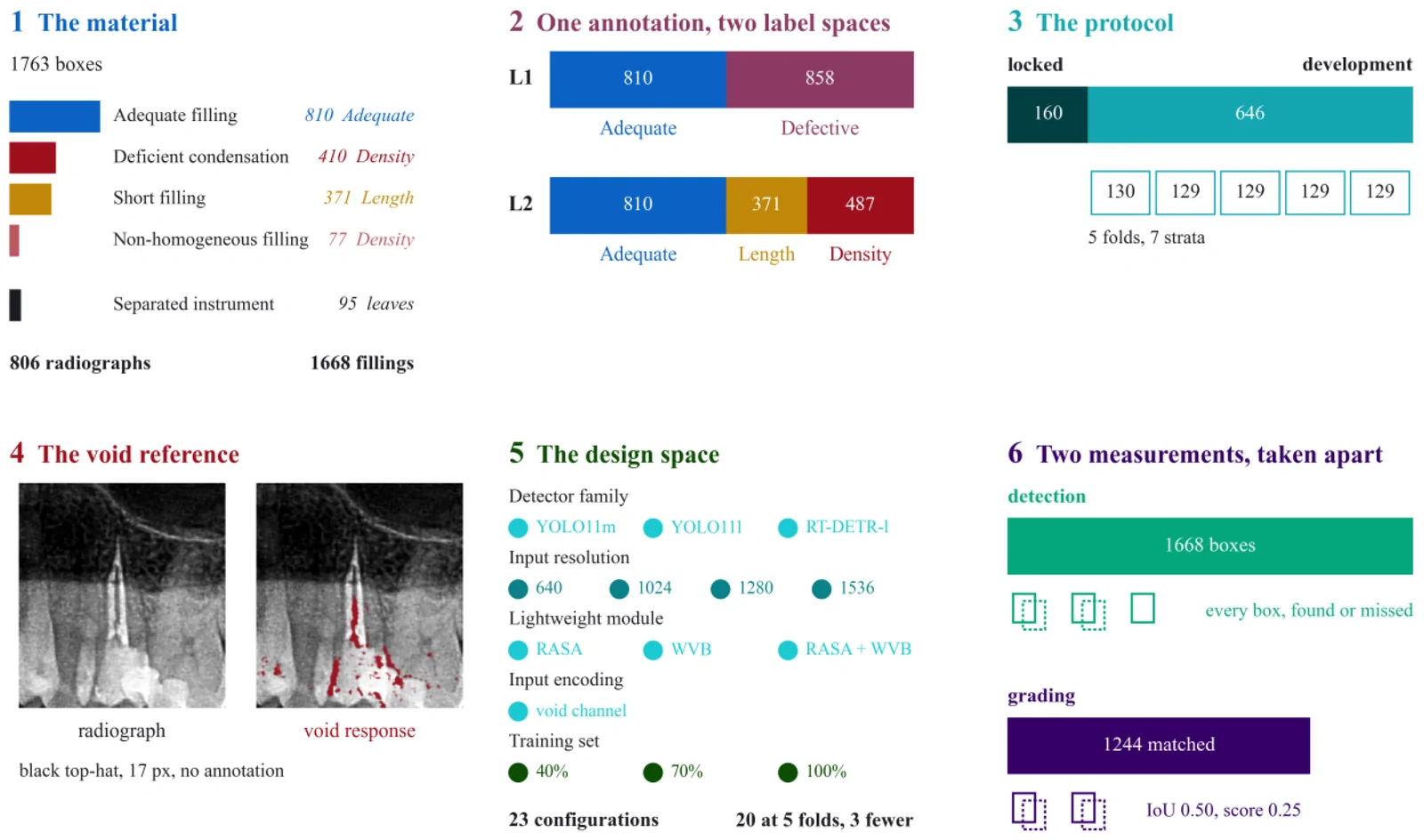
Overview of the study design, drawn as six numbered stages and reporting no outcome. (**1**) The material as collected, 1763 annotated boxes on 830 panoramic radiographs in five source categories, each bar measured against the largest of them and painted in the colour of the axis that category becomes. The separated instrument category is detached from the group and drawn in ink, because it is a procedural complication and not a quality criterion. Its 95 boxes leave the study, and they take with them the 24 radiographs that are left with no annotation. This reduces the material to the working set of 806 radiographs and 1668 root canal fillings. (**2**) The same 1668 fillings are partitioned twice, so that both bars run the full width and the adequate boundary falls at one position in each: the binary scheme L1 at 810 adequate against 858 defective, and the axis-resolved scheme L2 at 810 adequate, 371 length, and 487 density. The merged defective class of L1 is the two axes of L2 together, and it is neither of them. (**3**) For the allocation, a locked test set of 160 radiographs was held out before any model was trained. The development set of 646 is drawn in five folds stratified on seven annotation strata, and each fold validates on the radiographs drawn beneath the span it comes from and trains on the other 516 or 517. (**4**) The void reference, one density defect, and the brightness-gated black top-hat response are computed from the radiograph alone on a 17-pixel structuring element, with no annotation entering it. (**5**) In the design space, 23 fitted configurations, 20 of them over all five folds, two over three folds, and one over a single fold, are grouped by the lever family they belong to and drawn in that family’s colour. (**6**) For two measurements, taken separately, class-agnostic detection is read over every one of the 1668 reference boxes, found or missed. Matched-box grading is read only over the boxes that are prediction-matched at an intersection-over-union of 0.50 and a score of 0.25, which is 1244 boxes at the baseline configuration. Both bars start at one edge and share one scale, so the shortfall of the second is the quantity an average precision aggregates away. The glyphs beneath each bar are a reference box, solid, and the prediction over it, dashed.

The material and the design are set out in Figure 1. One annotation was read into two label spaces, a test set was locked away before training, and localisation and discrimination were measured separately. Attribution was scored against evidence computed from the radiograph, and that evidence was itself tested rather than assumed.

### 2.1. Dataset and Annotation

This retrospective, non-interventional study was approved by the Non-Interventional Research Ethics Committee of Firat University (approval number 2026/23-31, dated 17 September 2026) and follows the Declaration of Helsinki. Consent was waived, and images were anonymised at export. Radiographs were acquired for clinical indications at the Department of Oral and Maxillofacial Radiology of the same university between January 2023 and March 2025. A single unit was used (ProOne, Planmeca, Helsinki, Finland), at 60 to 70 kV and 2 to 7 mA with an exposure time of 2 to 10 s. Images were exported as JPEG at native resolution.

Examinations with at least one endodontically treated tooth and a visible root filling were eligible. An examination was excluded for poor image quality from blurring, distortion, or artefacts, for incomplete visualisation of the roots or the apical region, or for superimposition of anatomical structures that prevented reliable assessment of the root filling or the canal. Images were also excluded for extensive metallic restorations or artefacts that interfered with evaluation, or for severe overlap of teeth or roots. In all, 1129 consecutive examinations were screened, of which 212 were excluded on the quality criteria above. Of those that remained, 87 were later attendances by a patient whose earlier qualifying examination was already included, and these were set aside, since selection was one examination per patient, applied after the quality criteria. That leaves 830 panoramic radiographs. Each radiograph is therefore from a distinct patient, and no patient contributes to more than one partition. Each excluded examination is counted once, under its primary reason: poor image quality from blurring in 71, incomplete visualisation of the roots or the apical region in 44, superimposition of anatomical structures in 38, extensive metallic restorations or artefacts in 35, and severe overlap of teeth or roots in 24. The root fillings were placed with gutta-percha and an epoxy resin sealer (AH Plus, Dentsply DeTrey GmbH, Konstanz, Germany) using cold lateral condensation by the endodontics department of the same institution. Because radiopacity differs between sealer classes, the density criterion in Table 1 is read against that material and not against filling materials in general. The 830 patients, counted before the separated-instrument exclusion described below, had a mean age of 43.6 years, ranging from 18 to 81, and 448 were women. Of the 1668 fillings, 900, or 54.0%, have their box centre above mid-height of the film and are counted as maxillary, against 768 mandibular. The same reading by class gives 48.9% maxillary for adequate fillings, 50.1% for length defects, and 65.3% for density defects. Since the annotation carries no tooth number, the distribution over anterior, premolar, and molar teeth cannot be recovered from the boxes and is not reported.

Annotation was performed in ImageJ (version 1.53c; National Institutes of Health, Bethesda, MD, USA) by two specialist endodontists, each with more than ten years of experience. They read every radiograph independently of one another between 18 September 2026 and 23 September 2026. A third endodontist reviewed both readings and adjudicated every disagreement, and that adjudicated set is the reference standard used throughout. It is an expert-adjudicated reference read on the same panoramic projection rather than a measurement from an independent modality, and the terms “reference standard” and “reference box” are used in that sense. Only the adjudicated set was exported, because the annotation platform held one project file per cohort and the per-reader sessions were overwritten at that step. No inter-observer agreement can therefore be computed on this cohort. Since label noise bears directly on how grading performance is read, this is a principal limitation of the study. Every treated root was delineated with an axis-aligned box carrying one of five labels: adequate filling, deficient condensation, short filling, separated instrument, and non-homogeneous filling. These carry 810, 410, 371, 95, and 77 boxes, respectively, and 1763 in total. The label set follows the two requirements the European Society of Endodontology sets out for apical extent and for density. That report states them narratively, however, and gives no operational threshold for any of them, which is why the thresholds applied here are this study’s own and are stated in Table 1.

A calibration session preceded annotation. In it, the three readers independently scored forty panoramic radiographs that are not part of this cohort, and then discussed every disagreement until the criteria of Table 1 were applied consistently. Material extruded beyond the radiographic apex is not a label of this scheme. Twenty-nine roots carried such material. They were labelled on apical extent, according to the position of the main filling mass, regardless of what lay beyond the apex. A root whose main mass ended within 2 mm of the radiographic apex was labelled adequate, and one ending more than 2 mm short was labelled short. Of the 29, 21 were adequate, and 8 were short. The extrusion itself was not annotated, so no class in this scheme identifies it. The 21 labelled adequate carry no penalty for it, and the 8 labelled short are penalised for the position of their main mass only. Section 4.5 restricts the clinical claim accordingly.

Two of the criteria in Table 1 are stated in millimetres, and the images are stored in pixels, so the conversion is given here. The exported radiographs are sampled at 0.100 mm per pixel in the image plane, that is, at the detector and before any allowance for magnification. The manufacturer’s nominal magnification over the focal trough is 1.25, so one millimetre of object in the trough spans 12.5 pixels, and the 2 mm and 1 mm thresholds correspond to 25 and 13 pixels on the exported image at its native scale. The plane is stated because the two readings differ by the magnification factor itself: a spacing already referred to object space would put the two thresholds at 20 and 10 pixels instead. Panoramic magnification is anisotropic, so this is a single nominal factor and not a per-tooth calibration. Distances were measured along the canal axis at that scale, and no per-image correction was applied. These thresholds are therefore operational radiographic criteria applied on the panoramic image and not anatomical measurements. Magnification that varies across the image layer, distortion, and the superimposition of neighbouring structures all separate a distance measured on the film from the distance in the tooth, so a root close to either threshold could fall on the other side of it under a different projection.

A root meeting both the short criterion and a density criterion was assigned to the short filling class. A single-labelled scheme needs a precedence rule. Apical extent was given precedence because it was significantly associated with apical periodontitis in every cohort cited in Section 1, although density was associated as consistently and, in one cohort, more strongly. Thirty-seven roots met both. This rule has a consequence for what the two classes then contain, and it is stated here rather than left to be inferred. Every box remaining on the density axis is one whose apical extent was acceptable. The density class is therefore depleted of the roots that fail on both axes, which are plausibly the severest, and the length class carries them instead. Any comparison between the two axes in Section 3 is a comparison between a density class of length-acceptable roots and a length class that includes the doubly defective ones. The annotation does not record which of the short boxes these are, and Section 3.9 bounds the effect the opposite rule would have on the matched-box metrics.

Separated instruments were removed before training, since they are a procedural complication rather than a criterion of filling quality, even though they are a detection target in their own right [24]. Removing these 95 boxes left 24 radiographs with no annotation, and these were excluded too. The working set is therefore 806 radiographs and 1668 fillings. The separated-instrument roots on the radiographs that remained were not removed from the images.

**Figure 2:**
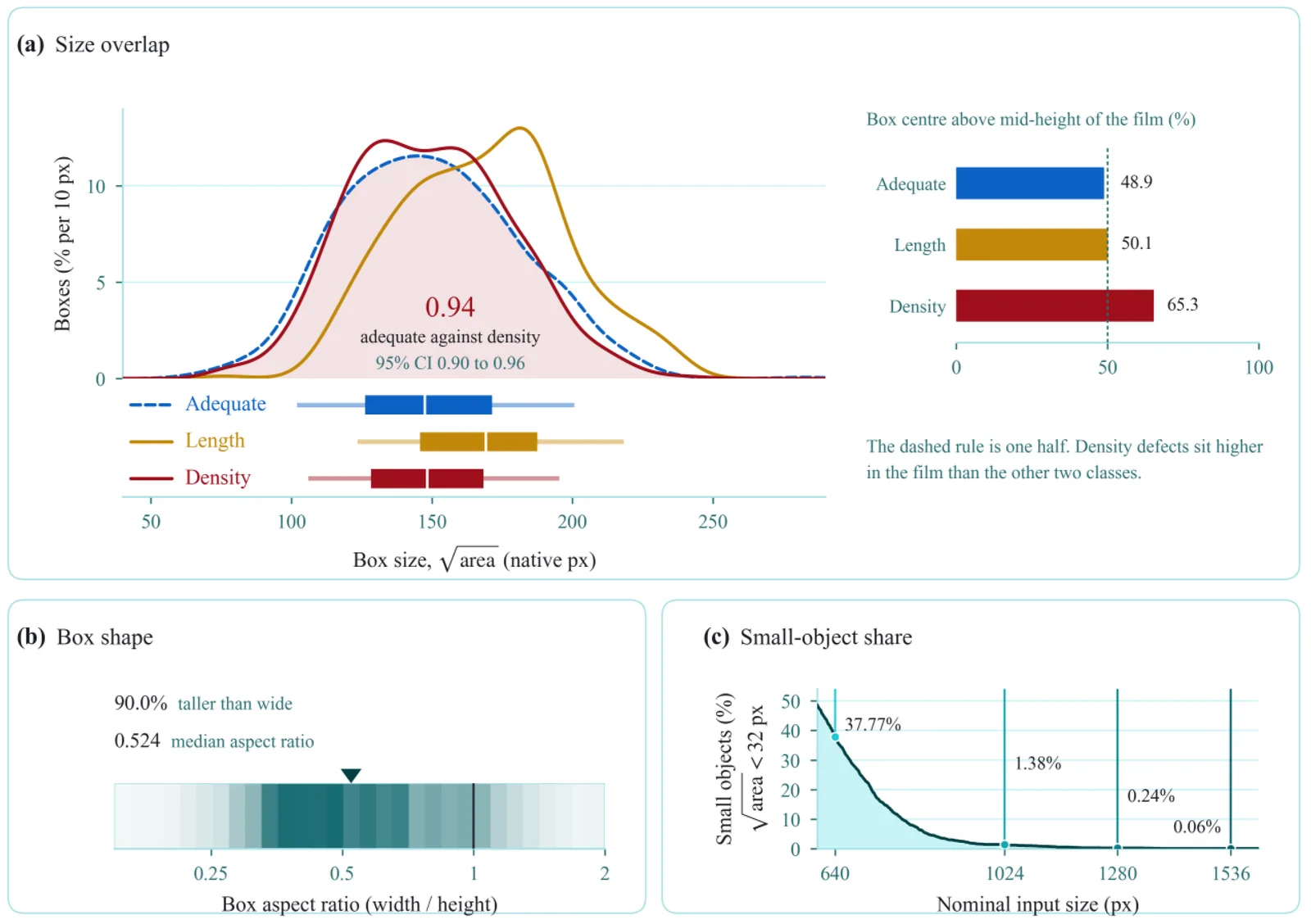
Geometry of the 1668 reference boxes on 806 radiographs, and what it predicts. (**a**) Class-wise box size as kernel densities of the square root of box area, with the mass the adequate and density classes hold in common filled. The overlap coefficient of that pair is 0.94, with a 95% bootstrap interval of 0.90 to 0.96 over 2000 within-class resamples, so size does not separate the two classes the dominant error runs between. The interquartile ranges beneath carry the same reading without an integral. The bars at the right give the share of box centres above mid-height of the film, 65.3% for density defects against 48.9% for adequate fillings. (**b**) Aspect ratio on a doubling axis, tinted by the density of boxes, 90.0% of them taller than wide with a median of 0.524, which is the morphology of a root. (**c**) Share of boxes under the COCO small-object threshold against nominal input size, with the four rungs of the resolution ladder marked. That share falls from 37.77% at 640 pixels to 1.38% at 1024, 0.24% at 1280, and 0.06% at 1536.

The boxes, drawn in Figure 2, are elongated vertically, with a median aspect ratio of 0.524 and a median square root of area of 152.9 pixels. Their class-wise sizes overlap almost completely, so the defect classes must be discriminated by appearance rather than scale. Reduction to the nominal input size leaves 37.77% of boxes below the 32-pixel small-object threshold of the Common Objects in Context (COCO) benchmark at 640 pixels, 1.38% at 1024 and 0.24% at 1280.

### 2.2. Nested Label Formulation

Two label spaces are derived from the same annotations, so the cost of finer clinical resolution is measured under an otherwise identical protocol. The binary scheme L1 merges all defect types into one defective class. The axis-resolved scheme L2 keeps adequate fillings apart and splits the remainder into a length axis and a density axis. The length axis holds short fillings. The density axis holds deficient condensation and non-homogeneous fillings, which the prevalence literature treats as one binary criterion rather than two. Both schemes are trained as flat detectors. They are nested so that every L2 class maps onto exactly one L1 class, which is what allows the two to be compared on the same boxes. The staged decision a clinician makes is therefore compared at its two levels rather than implemented as a hierarchy. Table 2 states both schemes.

### 2.3. Data Partitioning

A locked test set of 160 radiographs was held out before any model was trained. The remaining 646 form the development set, divided into five folds. Partitioning was at the image level, and since each radiograph comes from a distinct patient, it is also patient-level. Stratification uses the signature of each radiograph, which is the sorted set of distinct source classes it contains. Within each group, the radiographs are shuffled under a fixed seed. Then 20% go to the test set, with a floor of one for any group of at least three members, and the five folds take the remainder in rotation.

### 2.4. Detection Models and Training

YOLO11m is the primary architecture, because its cost allows a full five-fold protocol for every configuration [25]. RT-DETR-l enters as a transformer comparison, since its set-prediction formulation removes non-maximum suppression [26], and YOLO11l tests model scale. Resolutions of 640, 1024, 1280, and 1536 pixels were used, with 1024 as the reference for the architectural comparisons. All detectors were initialised from the framework’s COCO-pretrained weights. YOLO11l at 1280 pixels and the transformer at 1024 pixels completed three folds rather than five, and the wavelet module at 1536 pixels completed one. None of the three enters a comparison.

Experiments ran on one NVIDIA GeForce RTX 4090 (NVIDIA, Santa Clara, CA, USA) paired with an Intel Core i9-14900K (Intel, Santa Clara, CA, USA). The software was PyTorch 2.5.1 with CUDA 12.1, Ultralytics 8.4.90 and Python 3.10.13, together with pycocotools 2.0.11, NumPy 2.2.6, SciPy 1.13.1, and OpenCV 5.0.0. Convolutional detectors were trained for 60 epochs with early stopping at a patience of 20, whereas RT-DETR was trained for 120 epochs with a patience of 40, because transformers converge more slowly. A cosine schedule was used with AdamW at an initial learning rate of 0.001667, momentum 0.9, and weight decay 0.0005, with mosaic off for the final 10 epochs and the seed fixed at zero. Batch size ran from 16 at 640 pixels to 3 for RT-DETR at 1280 pixels, and gradient accumulation held the nominal batch at 64 throughout. Training the detectors reported here took 107 runs over 6261 epochs and 32.3 h on that one card. A run averaged 18.1 min, and the longest, RT-DETR at 1280 pixels, took 1.09 h. Three things sit outside that figure and are named rather than absorbed into it. Evaluation, augmented inference, fusion, and the attribution analysis ran from separate scripts that write no training log. The seven crop classifiers of Section 2.5.4 were trained without one, so their cost cannot be recovered. One further run crashed and was repeated, and the abandoned attempt accounts for 0.054 h that the total excludes. The figure is therefore a lower bound on the cost of reproducing the study rather than the whole of it.

### 2.5. Architectural Components Evaluated

Three components were evaluated against the 1024-pixel baseline: two lightweight modules, tested alone and together, and an input encoding. These are the four architectural interventions of Section 3.3. Each is given with its motivating reasoning, because none of them improved grading. The two modules sit before the detection head at the three pyramid levels, so the backbone architecture is identical across configurations.

#### 2.5.1. Root-Axis Strip Attention

Square kernels aggregate context isotropically, which dilutes the evidence that matters for a structure of median aspect ratio 0.524, since a length defect is defined by where the filling terminates along the root axis. Root-axis strip attention, RASA, aggregates the two image directions separately with depthwise strip convolutions of length 11, and then combines them convexly under spatially varying softmax weights. Its ancestor, the strip module of Strip R-CNN, applies horizontal and vertical strip convolutions in sequence under a single joint gate [27]. RASA instead weights the two directional branches separately, because the crown-to-apex direction inverts between the maxilla and the mandible. It adds 307,718 parameters, an increase of 1.5%.

#### 2.5.2. Wavelet Void Branch

Voids are low-contrast in the spatial domain, yet they are the defining content of the high-frequency subbands, which the wavelet void branch, WVB, makes available to the detection head. It decomposes each channel with a fixed, non-learnable Haar discrete wavelet transform of stride two, projects the low-frequency subband and the three high-frequency subbands separately with 1 × 1 convolutions, mixes them with a 3 × 3 convolution and reweights the result by channel attention. The branch is then added to the residual path through a single learnable scalar gate, tanh β with β initialised at zero, so the module begins fully closed and can stay closed if it is not needed. Earlier frequency-augmented detectors fuse a wavelet decomposition by multi-head attention [28] or reach the same information through learnable Gabor and adaptive high-pass filtering [29], with gating inside their fusion modules. The difference here is that the whole branch enters the residual path through one scalar initialised at zero, so the branch contributes nothing at initialisation. The gate does not measure the contribution of the branch on its own, because the output projection it scales is learned as well. In the ten per-fold models at 1024 and 1280 pixels, it settled at an absolute value between 0.04 and 0.08 at every pyramid level, 19 of the 30 gates positive and 11 negative, so the branch neither closed nor opened consistently. It adds 1,591,235 parameters, an increase of 7.9%, and the two modules together add 1,898,953, an increase of 9.5%.

#### 2.5.3. Native-Resolution Texture Encoding

A void of tens of pixels at an acquisition width of roughly 2800 pixels is nearly extinguished at 1024 pixels, whereas a texture statistic computed before the reduction survives it. Native-resolution texture encoding, NRT, therefore fills the two duplicate greyscale channels with statistics computed at acquisition resolution. The second carries the local standard deviation over a 9 × 9 window. The third carries a brightness-gated black top-hat response, taken with a 17 × 17 elliptical structuring element. That response is gated by a linear ramp on the local mean over a 61 × 61 window, rising from zero at the seventieth percentile, because dark texture in bone is not a void. The encoding is the only variable, and the third channel is reused as the objective reference in Section 2.8.

#### 2.5.4. Two-Stage Crop Classifier Used as an Oracle

A two-stage arrangement removes detector error, because the classifier sees only the crop a supplied box defines, and that crop is cut from the native-resolution image. A ConvNeXt-Tiny was initialised from ImageNet-22k pre-training and takes 224 × 224 crops with class-balanced sampling. It is trained on the reference boxes of each fold’s training images and evaluated on its validation images, which gives 1343 crops. This is an oracle, and it bounds what this classifier achieves when every crop contains the root filling it must grade. Because the detection head is scored on the boxes it matched, the two are compared on that common subset as well as on the full set of reference boxes.

### 2.6. Inference, Test-Time Augmentation, and Ensembling

Predictions are generated at a confidence threshold of 0.001 with a suppression overlap of 0.6, which preserves the low-confidence tail that average precision requires. Test-time augmentation was pure inference over trained weights through the framework’s multi-scale and horizontal-flip path, since flipping is anatomically admissible. Ultralytics 8.4.90 silently ignores the request for RT-DETR, which is therefore reported on plain predictions throughout.

Ensembling is by weighted box fusion [30]. Boxes are processed in descending score order into clusters C of the same category whose representative overlaps them by at least 0.55. The representative is the score-weighted mean of the members, as given in Equation (1),

(1) $$b_{\mathrm{cluster}}=\frac{\sum_{i\in C}s_{i}\;b_{i}}{\sum_{i\in C}s_{i}}$$

while the cluster score is the mean of the member scores, scaled by the number of member boxes relative to the number of fused models and capped at one, as given in Equation (2),

(2) $$s_{\mathrm{cluster}}={\bar{s}}_{C}\cdot \min\left(1,\;\frac{\left|C\right|}{M}\right)$$

where M is the number of fused models, so a box that few models support is discounted. Performance weighting is active for the reported ensemble. Before fusion, it multiplies the scores of model a by the weight given in Equation (3),

(3) $$w_{a}={\left(\frac{F_{1}^{\mathrm{macro}}\left(a\right)}{\max_{a\prime }F_{1}^{\mathrm{macro}}\left(a\prime \right)}\right)}^{2}$$

computed from the five-fold mean of its cross-validated matched-box macro-F1, where the square emphasises the difference between members. The scores entering Equations (1) and (2) are these weighted scores. Rank-based score calibration by percentile rank within each model is not active.

Both devices cost time, and the amounts are not comparable. In the measurements in Table 3, augmented inference close to doubles the cost, taking the baseline at 1280 pixels from 12.4 to 23.5 milliseconds and the wavelet configuration from 12.7 to 25.3, a factor of 1.9 to 2.0 in every case. Fusion itself is negligible by comparison. Only the per-model times are measured, and the cost of an ensemble is arithmetic on them: the three-member ensemble costs 70.8 milliseconds per radiograph with one-fold models of each member and 354 when all five are run, against 12.4 for a single unaugmented baseline at the same resolution, a factor of 28.7. Cost ratios quoted anywhere in this manuscript are formed on the Table 3 measure at both ends. Taken on the wall clock instead, the same comparison is 21.2 rather than 28.7, and mixing the two conventions inflates it further.

### 2.7. Evaluation Metrics

Three families of measurement are reported, because average precision alone penalises a wrong defect type exactly as it penalises a missed tooth.

The first is conventional COCO average precision over the reference standard, assembled from the YOLO labels using true image dimensions [31,32]. It is reported here as AP50. AP from 0.50 to 0.95, AP75, and the per-class values are held in the experiment ledger, which is deposited with the code in the public dataset described in the Data Availability Statement.

The second is class-agnostic detection quality, which answers whether the root filling is found at all. Detections above a score threshold of 0.25 are matched greedily to reference boxes on overlap alone, at a matching threshold of 0.50 intersection over union, with at most one detection per box.

The third is matched-box classification over those pairs. It is reported as accuracy, macro-averaged F1, balanced accuracy defined as the mean of the per-class recalls, and the confusion matrix. Because matching is class-agnostic, every pair is a correctly localised root filling, so confusion-matrix changes cannot be attributed to localisation. The two operating points differ deliberately: average precision requires the low-confidence tail at a confidence of 0.001, whereas the separated metrics require a usable operating point at a score threshold of 0.25.

Because separating the two families invites a reader to compare configurations on whichever of them flatters a method, the joint quantity is reported alongside them. It is the share of reference boxes that were both found and then graded correctly, the product of class-agnostic detection recall and matched-box accuracy, and the quantity a clinic would experience. All three are reported: the joint figure for what the system delivers, and the two components for where a difference between systems comes from.

### 2.8. Evidence-Grounded Attribution

Attribution needs a reference for where the evidence should be. A void in the narrow sense is a radiolucent discontinuity inside radiopaque filling material, and a brightness-gated black top-hat is an operator for that appearance. The third NRT channel therefore serves as an annotation-free void reference, shown beside its radiograph in Figure 1. Two qualifications belong here rather than in the discussion. The narrow sense is not the only one the European Society of Endodontology guidance carries, since that guidance also treats a general loss of radiopacity over the filled length as a density failure. Table 1, in turn, defines the larger density label, deficient condensation, by radiolucency at the filling/dentine interface, a generalised reduction in radiopacity or an interior void under 1 mm, and reserves a discrete interior lucency of 1 mm or more for the 77 non-homogeneous boxes. The operator is not that appearance, only a response to it. Section 3.6 measures how far the two fall apart, and shows the response separating on the deficient condensation label and not on the interior one. The reference is therefore a stand-in chosen because it needs no annotation, not a validated marker of voids.

Whether that signature is recoverable at all from the panoramic pixels was tested directly and without any model. Twelve descriptors were computed inside every annotated box. Six summarise the brightness-gated response itself, six describe the lucency inside the filling, four of them are connected-component measures, and two show the dispersion of grey level, among them the eroded interior depth contrast. Each descriptor was scored as a discriminator of density defects against adequate fillings and against length defects by the area under the receiver operating characteristic (ROC) curve, which gives twenty-four comparisons. Each was then tested against chance by the method of Hanley and McNeil with Bonferroni correction across all twenty-four. Because no model is involved, this analysis uses every annotated box of the working set; the boxes of the locked test set are included. After the merged result was in hand, the six gated-response descriptors were also scored against each density source label separately, against the same adequate fillings, with Bonferroni correction across the twelve comparisons of that split and, as a stricter reading, across all thirty-six. That split was not planned and is reported as post hoc.

What the operator itself responds to was measured separately and on synthetic images, because no annotated cohort holds lucencies of known size and shape at known positions. A uniform radiopaque strip 28 pixels wide, about 2.24 mm at the conversion of Section 2.1, was drawn on a 320 × 320-pixel dark background. A single lucency was then placed on it. Four arms were run, varying the position of the lucency, its size at fixed circular shape, its shape at fixed axial placement, and the width of the strip it sits in.

Detector randomised input sampling for explanation (D-RISE) needs only a mapping from image to detections, so it applies identically to both architectures [33]. Masks follow the RISE construction [34]: a binary grid whose cells are retained with probability p, upsampled bilinearly, shifted by a random offset of less than one cell and multiplied with the image. The cell side used here is 16 native pixels. Every detection under a mask is scored against the target by the similarity given in Equation (4),

(4) $$s_{i}=\max_{d\in D_{i}}\;\mathrm{IoU}\left(b^{*},\;b_{d}\right)\cdot c_{d}\cdot 1\left[{\hat{y}}_{d}={\hat{y}}^{*}\right]$$

where $D_{i}$ is the set of detections returned on the ith masked image, the starred quantities are the target box and predicted class, $c_{d}$ is the confidence of detection d, and the indicator restricts the maximum to the target class. A masked image that returns no detection of that class scores zero. Three hundred masks are used per target detection, at a confidence of 0.01. Over a film whose median size is 2800 × 1316 pixels. That budget places the attribution map in a subspace of at most three hundred dimensions, an effective spatial resolution of about 111 pixels against a median box side of 152.9 pixels, so the design resolves structure at the scale of the box and not at the scale of a void. It is raised to one thousand masks on the convolutional detector as a convergence check, which brings that figure to 61 pixels. The void region is the statistic that this budget is least able to resolve, because it is the upper quartile of the reference within the box and its ring. Attribution was computed on the plain baseline at 1280 pixels and on the transformer, neither of which receives the native-resolution texture encoding of Section 2.5.3, so the reuse of its third channel as the void reference introduces no circularity.

In a scripted probe, 16.4% of masks at the default retention of 0.5 recovered the target, against 82.1% at 0.9, so retention was fixed there. Every pixel is then visible in approximately 90% of masks, so the classical weighted sum is dominated by a near-constant offset. The attribution is therefore taken as the contrast on the similarity s between the masks that leave a pixel visible and those that occlude it, as given in Equation (5),

(5) $$A\left(x\right)=\frac{\sum_{i}s_{i}\;m_{i}\left(x\right)}{\sum_{i}m_{i}\left(x\right)}-\frac{\sum_{i}s_{i}\;\left(1-m_{i}\left(x\right)\right)}{\sum_{i}\left(1-m_{i}\left(x\right)\right)}$$

where $m_{i}\left(x\right)$ is the value of the ith upsampled mask at pixel x. Bilinear upsampling makes that value a weight between 0 and 1, so the two terms are visibility-weighted means of the similarity, and for binary masks, they reduce to its conditional means over the masks in which the pixel is visible and over those in which it is occluded. The contrast is exactly the first term minus the mean similarity over all masks, divided by the mean occlusion of the pixel over the masks. The offset it removes is therefore one constant per target, and only the divisor varies from pixel to pixel. It differs from the classical map with its spatial mean removed in that offset and in that divisor, which is why the two estimators compared in Section 3.6 agree closely but not to the digit. It is a correction for a near-saturated retention regime rather than a different estimand.

The interior is the target box. The ring is an axis-aligned rectangle of 1.5 times its extent, minus the interior. A wide field of 4 times the extent gives a coronal and an apical region, the parts of that field beyond the upper and lower edges of the box, each 4 box widths by 1.5 box heights. The arch decides which is which, the crown lying below the box in the maxilla and above it in the mandible. The two regions are equal in area, to within one pixel row, unless the edge of the film clips one of them, so the chance levels of the apical attribution overlap (AAO) and the coronal-to-apical ratio (CAR), both defined below, are 0.5 and 1. Let A be the attribution map, $a\left(x\right)$ its positive part, and B the void reference, whose upper quartile within the neighbourhood of the interior and ring defines the void region V, as given in Equation (6),

(6) $$\Omega =\mathrm{interior}\cup \mathrm{ring},\;V=\left\{x\in \Omega \;:\;B\left(x\right)\geq Q_{75}\left(B\;|\;\Omega \right)\right\}$$

Void attribution overlap (VAO) is the share of the positive attribution mass of Ω that falls in V, over the area share of V in Ω, which is the lift over chance, as given in Equation (7),

(7) $$\mathrm{VAO}=\frac{\sum_{x\in V}a\left(x\right)\;/\;\sum_{x\in \Omega }a\left(x\right)}{\left|V\right|\;/\;\left|\Omega \right|}$$

Writing m with a region subscript for the fraction of the positive attribution mass of the whole film that falls in that region, the remaining metrics are those given in Equation (8),

(8) $$\mathrm{AAO}=\frac{m_{\mathrm{api}}}{m_{\mathrm{cor}}+m_{\mathrm{api}}},\;\mathrm{CAR}=\frac{m_{\mathrm{cor}}}{m_{\mathrm{api}}},\;\mathrm{BRF}=m_{\mathrm{in}}+m_{\mathrm{ring}}$$

AAO is the counterpart of VAO for the length axis. CAR is a shortcut detector, above one when attribution shifts to the crown and its restorations. BRF, the box-and-ring fraction, reported in Section 3.6 as the mass inside the box and ring, measures whether the decision draws on local evidence. AAO and CAR are the same quantity in two readings, since CAR equals one over AAO minus one exactly. The two are therefore reported together for interpretability. The Holm family of the attribution suite nevertheless counts them separately, which makes that correction conservative. The pointing game [35] and its energy-based variant [36] are also recorded.

Every metric is computed identically for two mandatory baselines: a Sobel gradient magnitude for the null hypothesis that attribution tracks image edges, and a random smooth map for the hypothesis that any smooth map suffices. A metric on which D-RISE does not separate from both carries no evidence. That rule tests the estimator and not the reference. Where a metric returns no separation, the paired difference is therefore reported with the interval that bounds the excluded effect, and the reference itself is examined separately in Section 3.6. The suite is also repeated on a detector whose learned parameters have been re-initialised. That control cannot fail under the scoring function used here, so it is retained as a check that the pipeline is model-dependent end to end and not as a test that the attribution could have failed.

Detections are sampled and stratified into cells of predicted class crossed with correctness, 6 per cell in each of the 5 validation folds under a fixed seed, which gives 180 attributed detections per architecture at 1280 pixels. Paired comparisons of D-RISE against a baseline on the same detections use the Wilcoxon signed-rank test. Comparisons between independent sets of detections, the two architectures, correct against incorrect predictions, and two configurations attributed on their own detections use the Mann–Whitney U test. *p*-values are Holm-corrected within the suite. Intervals on mean differences come from 20,000 percentile bootstrap resamples of the detections, and the rank-pair effects drawn with the attribution results carry bias-corrected and accelerated intervals from 10,000 resamples. The test of the reference itself in Section 3.6 attributes the configuration that receives the native-resolution encoding and the plain baseline at 1024 pixels, on 180 detections each, sampled the same way.

### 2.9. Statistical Analysis

Every configuration is trained under five-fold cross-validation on the 646 development radiographs, with fold variation reported as the mean and standard deviation of the five values. Comparisons are not made on those fold means: the paired fold differences carry a standard deviation of 3 to 9 points, so at a sample size of five, that design would resolve only effects of around 11 points. They use instead a pooled image-level paired bootstrap, in which the out-of-fold predictions of all five folds form one evaluation set containing every development radiograph once, 646 are drawn with replacement, both configurations are evaluated on the same resample, and every metric is recomputed from scratch. The reported interval is the 2.5th to 97.5th percentile of the merged difference distribution, and the two-sided *p*-value is twice the smaller of the proportion of differences at or below zero and the proportion at or above zero, capped at one. Every configuration comparison uses 2000 resamples, so the finest two-sided *p*-value it can express is 0.001, and no smaller value is reported for it. The rank tests of the attribution suite are not bound by that floor. The comparison of the two label spaces resamples the same 646 radiographs under both schemes, since the folds of L1 and L2 hold identical images.

The resampling draws images only, since the fitted models and their stored predictions are held fixed. Every planned interval reported here is therefore an estimate of test-set sampling variability conditional on one training draw, and it does not contain training stochasticity. Section 4.4 reports what a second seed did to one configuration.

Each comparison yields up to nine metrics from one resampling, and no primary endpoint was designated in advance, so *p*-values within a comparison are corrected by the Holm procedure across that comparison’s own metrics. The correction changes the reading of seven comparisons over twelve metrics, each a result this manuscript would otherwise have claimed. Both the uncorrected and the corrected value are printed where Section 3 draws on one of them, and the corrected value alone elsewhere. Fifteen such comparisons were made, and no correction is applied across them, because they are treated as separate questions asked of the same development set rather than as one family. A reader who prefers a stricter policy therefore has the count and the uncorrected values needed to apply one. The descriptor search of Section 2.8 is Bonferroni-corrected across its own twenty-four comparisons, and the attribution suite is Holm-corrected across its own.

Two candidate ensembles were built on the development folds, a two-member and a three-member subset. The folds did not separate them, at 66.46 against 66.22 macro-F1 with the two-member subset nominally ahead. The final measurement involves no retraining, since validating a refitted model on the test set would let the framework select a checkpoint by looking at it. The cross-validation models are applied unchanged to the 160 held-out radiographs in two passes, one axis-resolved and one binary. The test set entered no training loop and no checkpoint selection. It was consulted for the choice between the two candidate ensembles, and it chose the three-member subset. The macro-F1 reported for that ensemble is therefore a maximum over two candidates and carries the optimism that implies. Ten binary readings were also scored on the test set, and the one whose operating characteristics are reported is the highest of them.

### 2.10. Post Hoc Subgroup Analyses

Two subgroup analyses are reported below. Both were run after the results above were in hand, and neither was planned. They are drawn from a wider exploratory examination of where the axis-resolved errors fall, which included splits by sextant, by image zone, and by acquisition cluster. That examination is not reported here, and none of its subgroup estimates enters this manuscript. One of its findings, an association between acquisition cluster and class prevalence, bears on the limitations and is reported in Section 4.4 with its test statistic as an exploratory result. The two subgroup analyses are reported because the manuscript prints a positional imbalance it would otherwise leave uninterpreted. They are labelled here so that no reader takes them for pre-specified findings. Neither enters the Holm families of Section 2.9, which cover the planned configuration comparisons, and no claim in the Abstract or the Conclusions rests on either. The descriptor split of Section 2.8 is post hoc in the same sense, and the Conclusions label it as such where they draw on it.

The first splits the matched boxes by jaw, a box counting as maxillary when its centre lies above mid-height of the film, which is the rule Section 2.1 uses. It is reported with both error arms, because a single arm of a two-class confusion moves with the base rate of the class, and with the Youden index, which does not. The second returns the matched density boxes to the two source labels of Table 1, deficient condensation and non-homogeneous filling, and compares how often each is missed. A matched density box counts as missed when the model returns it as an adequate filling. That is one off-diagonal cell and not the complement of sensitivity on the class, because a density box returned as a length defect is a different error and is not counted here. Pooled over both source labels, the rate so defined is 29.3% of the 365 matched density boxes, 107 of them. The complementary arm cannot be formed for this split, since the source labels exist only on density boxes, so the second analysis is one-armed by construction and is read only against the other source label, never as a statement about how the two are separated. Both use the matching rule in Section 2.7 and the radiograph-clustered resampling in Section 2.9 over 2000 draws, the unit resampled being the radiograph. The universe differs between them, because each is restricted to the development films carrying a matched box of the relevant kind: 618 radiographs for the jaw split at 1024 pixels, 620 for the same split at 1280, 610 for the subset both resolutions read, and 267 for the source-label split.

### 2.11. Sensitivity Analyses

Four sensitivity analyses were added after peer review. All of them reuse the stored predictions, with no retraining and no further model selection, and all are post hoc in the sense of Section 2.10. The first concerns the 71 separated-instrument roots left without a label on the radiographs that were retained. Their boxes were taken from the source annotation and scored in three ways: how many of them the detector covered under the matching rule, how detection precision and F1 change when the detections lying on them at an intersection over union of at least 0.50 are ignored rather than counted as false positives, and whether detection recall on the reference boxes of the radiographs that carry such a root differs from recall on the other radiographs, under a radiograph-level bootstrap of 2000 resamples. The second concerns the 37 roots that met both the short criterion and a density criterion and were assigned to the short class. The annotation does not record which 37 of the 371 short boxes they are, so a direct re-analysis is not possible. The matched-box metrics were instead bounded under the opposite precedence rule by its two extreme assignments, with the model held fixed: all 37 taken from short boxes the model called density, which is the most favourable case, and all 37 from short boxes it called short, which is the least favourable. Because all 37 could lie in the development set, the bound is computed there at its full size. The third counts end-to-end sensitivity directly on the locked test set, with every defective filling the detector did not find being counted as a false negative. The fourth reports every binary reading scored on the locked test set rather than only the highest, together with the reading the development folds would have selected, the three-member binary ensemble, whose cross-validated macro-F1 of 77.58 was the highest of any binary configuration.

## 3. Results

Every configuration was trained and evaluated under five-fold cross-validation on the 646 development radiographs. No model-based value reported before Section 3.7 comes from the locked test set. The box geometry of Section 2.1 and the descriptor analysis of Section 3.6 involve no model and use every box of the working set. Table 4 collects the three families of measurements in Section 2.7, reporting detection recall and classification accuracy separately.

### 3.1. The Cost of Refining the Label Space

At 1024 pixels under an otherwise identical protocol, class-agnostic detection recall was 93.1% under L1 and 92.6% under L2 as five-fold means, and 92.93% against 92.63% pooled. That is a difference of 0.30 points with an interval of minus 0.99 to 1.70 at an uncorrected *p* of 0.693 and a corrected *p* of 1.000. Matched-box classification accuracy, by contrast, fell from 75.13% to 63.72% as five-fold means and from 75.16% to 63.75% pooled, a loss of 11.41 points with an interval of 8.20 to 14.46 at *p* below 0.001. Pooled macro-F1 fell with it, from 74.98 to 61.47, a loss of 13.51 points with an interval of 10.22 to 16.68 at *p* below 0.001. Class-agnostic detection F1 fell by 1.54 points, over an interval of 0.19 to 2.87 at an uncorrected *p* of 0.028, but that difference does not survive the correction of Section 2.9, at a corrected *p* of 0.112, and is therefore not claimed. Collapsed onto the binary decision, the axis-resolved predictions give 74.04% against 75.16%, a difference of 1.12 points with an interval of minus 1.33 to 3.53 at an uncorrected *p* of 0.408 and a corrected *p* of 1.000. The residual cost of assigning the axis, by contrast, is 10.29 points with an interval of 8.48 to 12.18 at *p* below 0.001. Almost all of the loss is therefore the cost of the axis assignment itself rather than a degradation of the binary decision. Every interval in this paragraph comes from a cross-schema image-level paired bootstrap over the 646 development radiographs, the two label spaces holding identical folds, at 2000 resamples.

**Figure 3:**
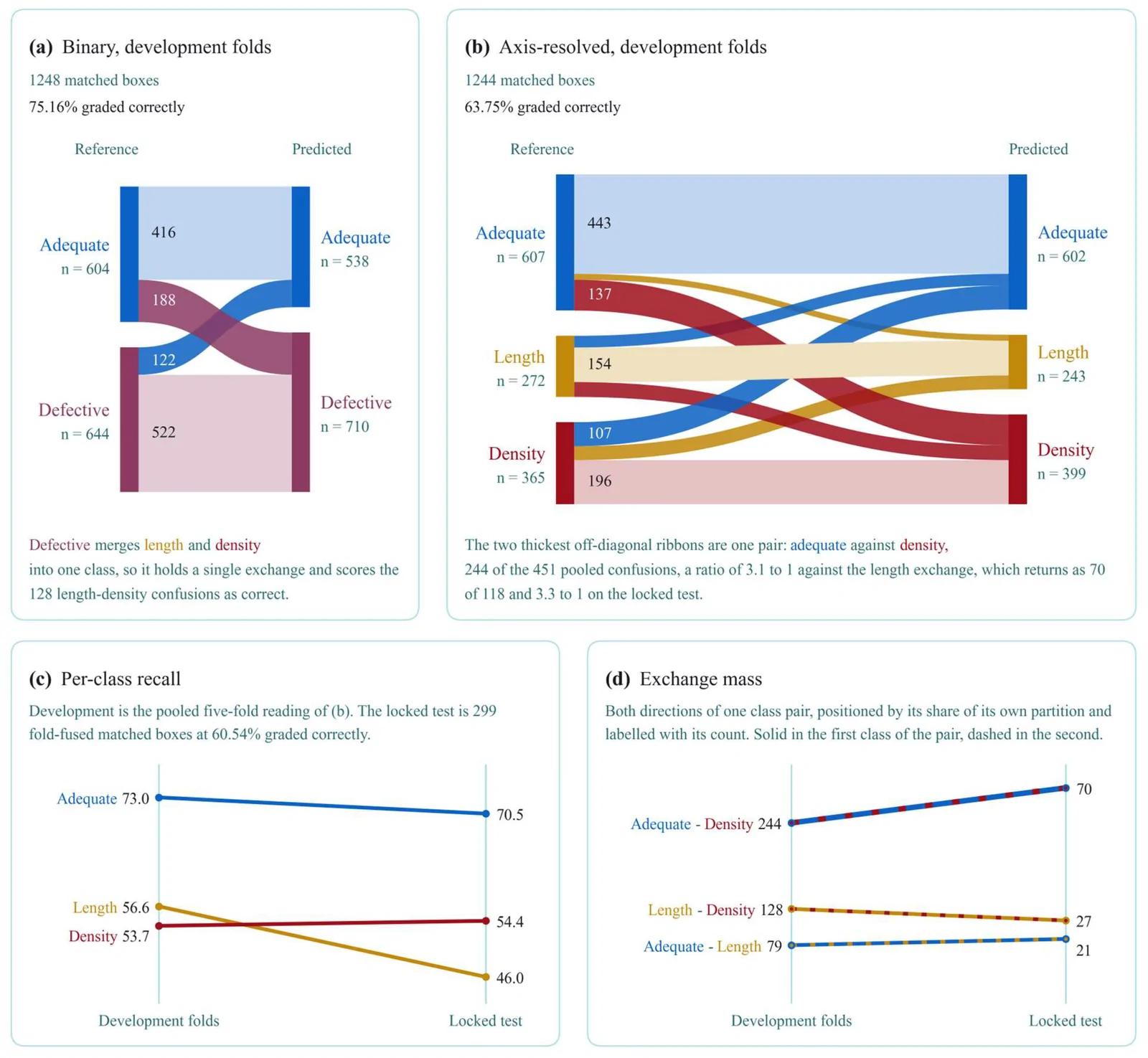
Where the error lands when the label space is refined from two classes to three, drawn as flow rather than as tables. (**a**) The binary scheme over the five development folds, 1248 matched boxes at 75.16% classified correctly. Reference classes stand at the left and predicted classes at the right, and ribbon thickness is the number of matched boxes. Its defective class merges length and density, so it holds a single exchange and scores as correct the length/density confusions that panel (**b**) counts. (**b**) The axis-resolved scheme over the same folds at the same scale: 1244 boxes at 63.75% classified correctly. Both percentages are matched-box classification accuracy and not the joint quantity Table 4 reports. The two thickest off-diagonal ribbons are one pair, adequate against density, carrying 244 of the 451 pooled confusions, a ratio of 3.1 to 1 against the length exchange. (**c**) Per-class recall from the development folds to the locked test: 73.0%, 56.6%, and 53.7% against 70.5%, 46.0%, and 54.4% over 299 fold-fused matched boxes at 60.54%. (**d**) The mass of each class-pair exchange as a share of its own partition: 244, 128, and 79 confusions on the folds against 70, 27, and 21 on the locked test. The dominant exchange returns there at 3.3 to 1.

Figure 3 draws the pooled confusion matrices, whose accuracies are 75.16% and 63.75% and whose axis-resolved recalls are 73.0%, 56.6%, and 53.7%. Counting both directions, adequate fillings and density defects are confused 244 times, the length and density axes 128 times, and adequate fillings and length defects 79 times. Refining the label space therefore leaves detection recall where it was and places the cost on discrimination. Those 244 exchanges do not fall evenly on the two jaws, and the post hoc split of Section 2.10 shows why the pattern is not what it first appears to be. A density defect is returned as adequate in 45.0% of mandibular boxes against 21.6% of maxillary ones, a difference of 23.4 points over a radiograph-clustered interval of 13.3 to 33.0 at a *p* below 0.001. The mirror arm runs the other way: an adequate filling is returned as a density defect in 31.3% of maxillary boxes against 15.2% of mandibular ones, 16.1 points over an interval of 9.4 to 23.0 at a *p* below 0.001. Both arms are properties of one operating point applied to two populations with different base rates, since density accounts for 46.8% of the matched adequate and density boxes in the maxilla and 26.7% in the mandible. Separating the two effects therefore needs a measure that does not move with the base rate. Take the adequate against density decision as a two-by-two over the boxes the model assigned to one of those two classes, so that the index does not depend on which of them is called positive. On that reading, the Youden difference between the jaws is minus 0.104 against the mandible, over an interval of minus 0.233 to 0.033 at a *p* of 0.132, an interval that contains zero.

At 1280 pixels, the two error arms move further apart, to 30.1 and 21.1 points, and the difference reaches minus 0.194 over an interval of minus 0.326 to minus 0.064 at a *p* of 0.003, an interval that does not contain zero. That the contrast resolves at one resolution and not at the other is not evidence that the two resolutions differ. Restricted to the 610 radiographs, both resolutions read the jaw difference as 0.118 against the mandible at 1024 pixels and 0.194 at 1280. The paired difference between those two is minus 0.076, with an interval of minus 0.213 to 0.070 at a *p* of 0.285, containing zero. The 1024 component is not the 0.104 given above, because that figure is computed on the full matched set at that resolution rather than on the common subset. The 1280 component rounds to the same 0.194 as the figure above, although the two values differ in the third decimal, because the two matched sets at that resolution are almost the same films. What the arch does at both resolutions is move where the errors fall. Whether it also costs separation is left open, and both readings are post hoc under Section 2.10.

One split does separate. Return the matched density boxes to the two source labels they were drawn from: deficient condensation and non-homogeneous filling. The arch effect is present in the first, at 28.8 points over an interval of 17.6 to 39.4 at a *p* below 0.001. It is not detected in the second, at minus 3.2 over an interval of minus 31.8 to 25.7 at a *p* of 0.837. The difference between those two is not itself tested, and the second interval is 57 points wide on 42 maxillary against 18 mandibular boxes. What the pair supports is therefore an effect established on one label and undetermined on the other rather than an interaction between them. Within the maxilla alone, the discrete-void label is missed 31.4 points more often than the diffuse one, over an interval of 15.8 to 48.3 at a *p* below 0.001. Both source labels are enriched in the maxilla to the same degree, 66.6% against 70.0%, so this is not the arch imbalance in another guise. The subset the detector reads worst is 60 of the 365 matched density boxes, and it is the one Table 1 defines by discrete radiolucent interruptions within the body of the material. Because the void reference of Section 2.8 is defined on a discontinuity inside the radiopaque material, that is the appearance the reference is built on. The detector is therefore weakest on the 60 boxes the reference is named for, 16% of the 365 matched density boxes, and strongest on the 305 assigned to deficient condensation. Section 4.2 measures what the reference actually responds to. A size account cannot be tested lesion by lesion there, because the annotation records a void by its longest dimension while the operator responds to its shortest, and, conditional on the difference between the two labels being real, a positional account of it is excluded. Both shares are of the matched boxes rather than of the 487 density boxes in the annotation, which divide 410 to 77 on the same distinction.

### 3.2. Input Resolution and Training Set Size

For the plain baseline under the axis-resolved label space, macro-F1 rose from 54.88 at 640 pixels to 60.90 at 1024 and 64.74 at 1280, then stood at 64.05 at 1536. The ladder therefore rises to 1280 and does not rise further within the resolution of a single-seed design. Those four values are five-fold means, and the two adjacent steps subtract to 3.83 and minus 0.69 as fold means. Read as pooled bootstrap estimates, which is the form the intervals belong to, the step from 1024 to 1280 raises macro-F1 by 3.51, over an interval of 0.45 to 6.55 at an uncorrected *p* of 0.029. That does not survive the correction of Section 2.9, at a corrected *p* of 0.203. The step from 1280 to 1536 lowers it by 0.90 over an interval of minus 3.67 to 1.91 at an uncorrected *p* of 0.554. Since neither step of the ladder is established under the declared policy, the rise to 1280 is a consistent ordering across four rungs rather than a resolved difference, and no plateau is claimed from the turn at 1536. A second seed at 1280 pixels, reported in Section 4.4, does not preserve that ordering, so 1280 pixels is read throughout as the highest rung of a single-seed ordering and not as an established optimum. On the density axis, neither step is resolved, the two differences being 2.65 and minus 2.66 over intervals that contain zero. The rise is carried by classification accuracy, 58.84% to 67.49%, not by detection recall, 90.8% to 92.2%.

**Figure 4:**
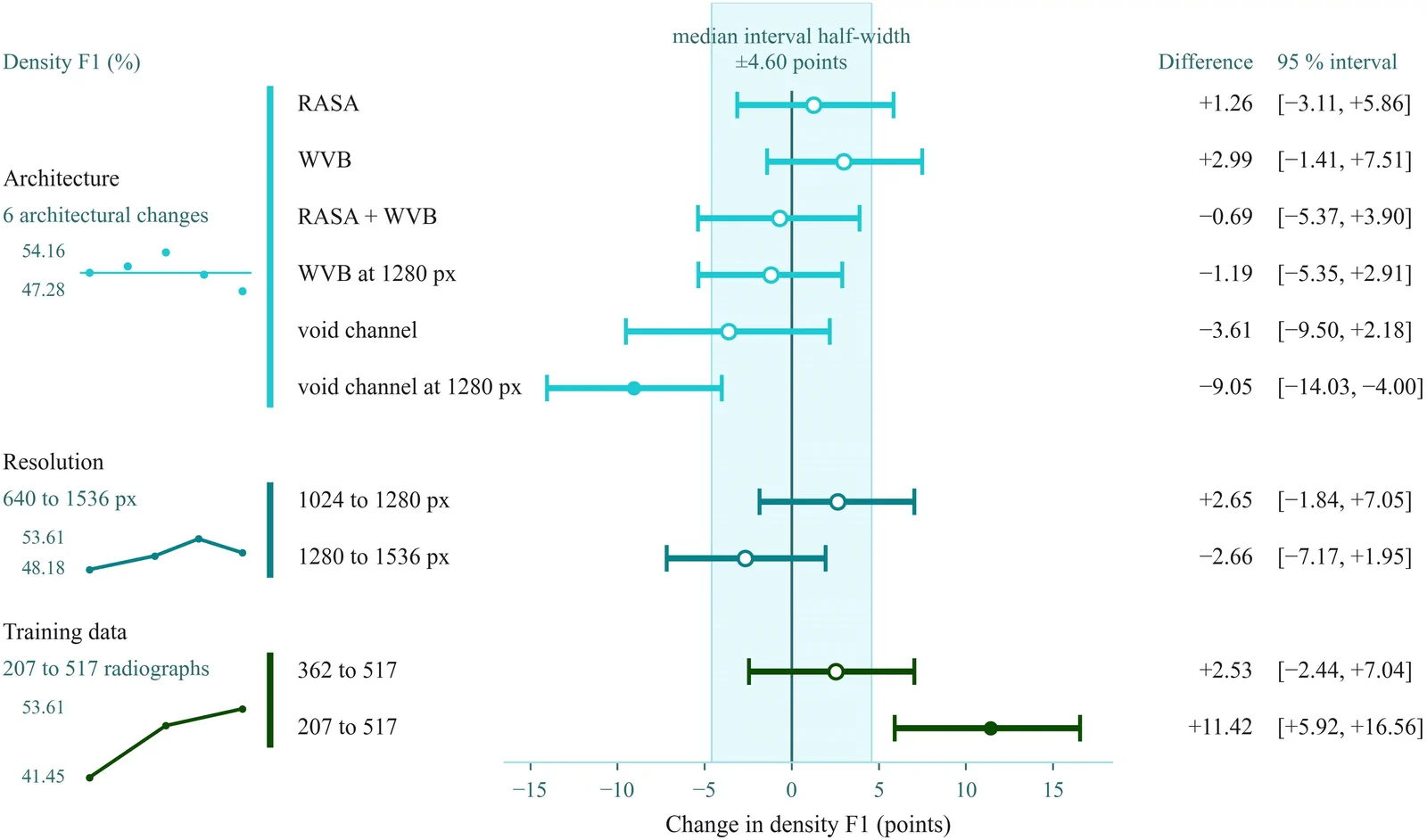
Three levers on the density axis, and which of them the resampling can resolve. The forest plot reports every stored pooled image-level paired bootstrap over the 646 development radiographs that bears on one of the three levers. Each one is drawn as a difference in density F1 with its 95% confidence interval, grouped by lever and bracketed in the colour of the sparkline beside it. The vertical rule marks zero effect. The shaded band marks the median half-width of the ten intervals drawn, plus or minus 4.60 points. That half-width corresponds to a minimum detectable difference of 8.48 points at 80% power on the density axis under the Holm-corrected alpha of Section 2.9, or 6.57 at an uncorrected alpha, and the per-comparison values are given in Section 3.3. A density F1 difference narrower than that cannot be told from zero at this sample size on this metric, whatever its point estimate. Two comparisons clear both the rule and the band and carry filled marks. Raising the training set from 207 to 517 radiographs per fold gains 11.42 points, over a 95% interval of 5.92 to 16.56, and the void channel retested at 1280 pixels loses 9.05 points, over an interval of minus 14.03 to minus 4.00. Six architectural comparisons are drawn. The four architectural changes at 1024 pixels give point estimates from −3.61 to +2.99, over intervals running from −9.50 to +7.51. The wavelet module and the void channel retested at 1280 pixels give −1.19 and −9.05, each read against the baseline at its own resolution, which is why the two 1280-pixel rows do not share the 1024-pixel reference. The two adjacent steps of the resolution ladder give +2.65 and −2.66, and the last step of the learning curve gives +2.53. Every one of these intervals contains zero except the void channel at 1280 pixels, whose interval is given above and is one of the two filled marks. Every comparison drawn is a pooled image-level paired bootstrap over the same 646 radiographs at 2000 resamples. The sparklines show the shape of each lever on one shared vertical scale of density F1, with each lever’s own extremes printed in its gutter. At 1024 pixels, the architecture scatters between 47.28 and 54.16 about its baseline. The resolution ladder rises from 48.18 at 640 pixels to 53.61 at 1280 and turns down to 51.17 at 1536, and the learning curve climbs from 41.45 at 207 radiographs to 53.61 at the full development set without turning.

The training partition of each fold was then reduced to 40% and to 70%. At 207, 362, and 517 training radiographs, the density axis reached five-fold mean F1 values of 41.45, 50.64, and 53.61, while macro-F1 stood at 55.73, 61.30, and 64.74, drawn in Figure 4. Over its whole range, the curve has not flattened: the density gain from the smallest to the largest partition is 11.42 as a pooled bootstrap estimate, over an interval of 5.92 to 16.56 at *p* below 0.001, against a difference of fold means of 12.16. Training set size is therefore one of the two manipulations of the data whose effect on grading the resampling resolves. Section 3.3 reports a third resolved effect, but it is a change to the input encoding rather than to the training data, and it runs against the density axis. Whether the curve is still climbing at the top of the range is a separate question, and this design does not answer it. The last step, from 362 to 517 radiographs, gains 2.53 points over an interval of minus 2.44 to 7.04 at a corrected *p* of 0.692. The reading rule of Section 2.9 therefore does not let that step be claimed. The second manipulation is mosaic augmentation. It raises pooled macro-F1 from 60.73 to 64.98, a gain of 4.24 points with an interval of 1.38 to 7.23 that survives the correction of Section 2.9 at a corrected *p* of 0.027. It also raises density F1 by 5.13 points with an interval of 0.96 to 9.36, which does not survive at an uncorrected *p* of 0.019 and a corrected *p* of 0.114. Both are levers on the data rather than on the model.

### 3.3. Architectural Components

One reading rule is applied throughout this section and Section 3.2. A difference is reported as established only if its interval excludes zero and its *p*-value survives the correction of Section 2.9, whatever its sign and whatever argument it would support. The interval is printed beside it, so that the reader may judge the size of what is not established. Where the two criteria disagree, the corrected *p*-value governs.

Of the four architectural interventions against the 1024-pixel baseline, root-axis strip attention returned an AP50 difference of −0.14, at an uncorrected *p* of 0.847 and a corrected *p* of 1.000. Its detection F1 difference was −2.26 over an interval of −3.62 to −0.90, at an uncorrected *p* of 0.002 and a corrected *p* of 0.018. The wavelet void branch raised macro-F1 from 60.90 to 63.56 at 1024 pixels as five-fold means, a pooled difference of 2.25 over an interval of minus 0.85 to 5.59, at an uncorrected *p* of 0.151 and a corrected *p* of 1.000. At 1280, it lowered macro-F1 from 64.74 to 63.75. Neither movement is established by the bootstrap. Combining the two modules dropped detection recall to 90.2%, the lowest of any configuration in Table 4, and native-resolution texture encoding produced no coherent gain.

Read together, the four give one clean result. None is separable from the baseline on macro-F1 or on the density axis, the four density intervals spanning −9.50 to 7.51 and every one containing zero. Two of them degrade class-agnostic detection at a level that survives correction, at corrected *p*-values of 0.018 and 0.009, and both of those two contain the strip attention module. The input encoding was also trained at 1280 pixels and compared with the plain baseline there under the same pooled bootstrap. It does not raise macro-F1: the difference is 2.52 points against it over an interval of minus 5.56 to 0.60 at a corrected *p* of 0.530. On the density axis, it is worse by 9.05 points over an interval of minus 14.03 to minus 4.00 at a corrected *p* of 0.009. The encoding therefore does not merely fail to help at the highest rung of the resolution ordering; it costs the axis it was designed to serve, while raising class-agnostic detection F1 by 2.07 points over an interval of 0.73 to 3.35 at the same corrected *p*. Both resolved differences share one corrected *p* because both raw values sit at the resampling floor of 0.001, and the monotonicity step of the Holm procedure then assigns the second the adjusted value of the first.

What that establishes is bounded by what the design can resolve. At the standard deviations these four comparisons carry, the smallest density-axis difference the resampling would detect on four occasions in five lies between 8.22 and 10.77 points, and on macro-F1 between 5.66 and 6.50 points. These are therefore non-detections of effects smaller than that rather than demonstrations of no effect. Those floors are computed at the threshold at which the decision rule of Section 2.9 actually applies, the Holm-corrected alpha of 0.05 over nine metrics rather than an uncorrected 0.05. That is the stricter and therefore the safer figure for a negative finding. At an uncorrected alpha, the same intervals give 6.37 to 8.35 and 4.39 to 5.04. The standard error behind both is recovered from the width of the percentile interval, which assumes a symmetry the resampling itself does not, an approximation adequate at these widths. Because the floor is recovered from the same interval widths it is then compared against, it is a monotone transform of them. Under the Holm alpha, it is about 1.85 times the half-width. Saying that an effect falls below the floor is therefore close to saying that the point estimate sits within about five sixths of a half-width of zero. The floor is therefore a restatement of how precisely this design measured these comparisons rather than an independent property of the design, and the margins are thin: on macro-F1 the largest upper bound falls below the smallest floor by less than a tenth of a point. That is why the claim is put as the absence of an improvement larger than the floor and never as the absence of an effect. The macro-F1 floor is above the 4.24-point effect of mosaic augmentation reported in Section 3.2, and by a wider margin than the uncorrected figure suggested. This design could therefore not reliably have detected an effect the size of the one it does resolve, and the architectural null should be read against that rather than as a demonstration that these modules do nothing.

Two augmentation configurations complete the inventory of what was run to five folds. Turning mosaic augmentation off at 1280 pixels lowers pooled macro-F1 from 64.98 to 60.73, which is the comparison reported in Section 3.2. A rectangular 1856-pixel input without mosaic reaches 62.45 pooled macro-F1 against that same 60.73, a gain of 1.71 over an interval of −1.33 to 4.77 that the resampling does not resolve, while its detection recall falls by 1.64. The rectangular run is a null and is reported so that the inventory reads as complete rather than as selected.

**Figure 5:**
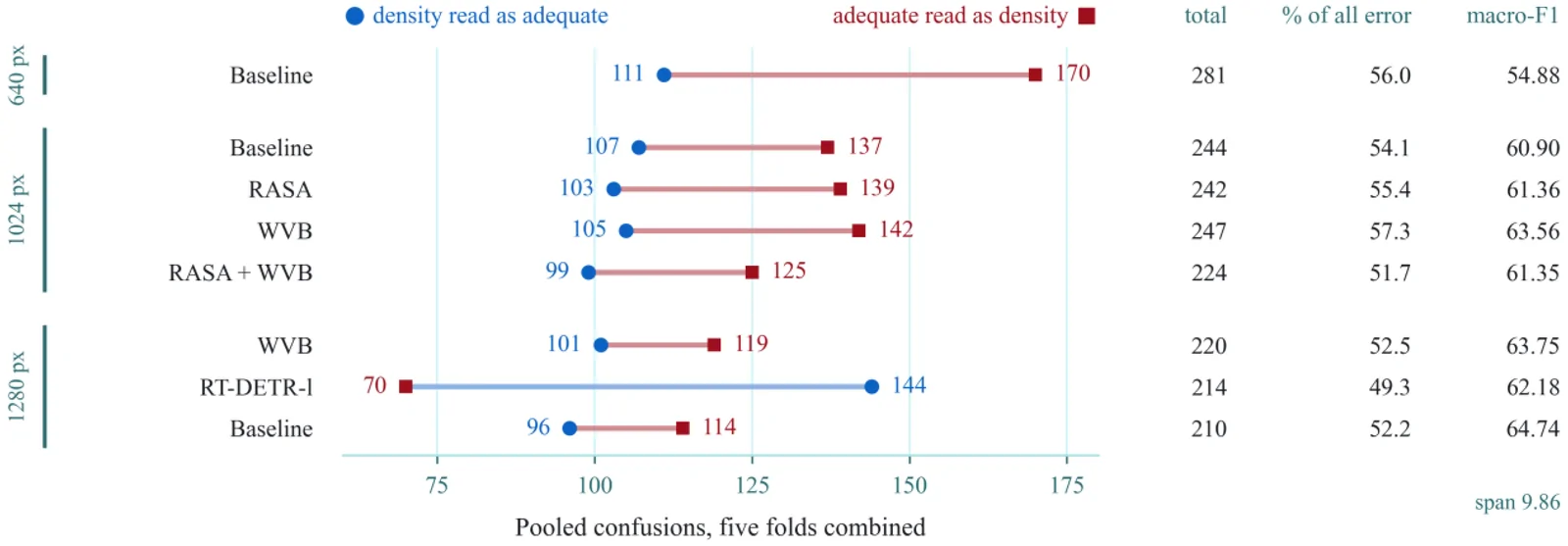
The exchange between adequate fillings and density defects, in both of its directions, for the eight axis-resolved configurations named in Section 3.3. Four further five-fold configurations are listed there and are not drawn. Each row is one configuration, and its two ends are the two directions of that exchange, set on one count axis and coloured by the decision that the model made. The adequate end is therefore the density defect the model read as adequate, and the density end is the adequate filling it read as a density defect. Rows are bracketed by nominal input size, and the pairs drift left as that size rises. At the 1024-pixel baseline, the exchange runs 137 adequate fillings read as density defects against 107 density defects read as adequate. The set-prediction transformer is the one row whose ends swap sides, at 70 against 144, which is a redistribution of the exchange and not a reduction of it. The three columns on the right give the total of the two directions, that total as a share of every misclassification the configuration makes, and the five-fold macro-F1 of the same configuration. Across the eight, the total holds between 210 and 281 counts and between 49.3% and 57.3% of all misclassifications, while macro-F1 spans 9.86 points. Architecture therefore moves the score and rearranges the exchange within the shelf that its input size sets. Among the drawn configurations, input resolution is the only change that moves the shelf, 224 to 247 counts at 1024 pixels against 210 to 220 at 1280. The mosaic-free run at 1280 pixels, listed in Section 3.3 and not drawn, sits above that band at 241. All counts are pooled over the five development folds.

Figure 5 shows the adequate against density exchange redistributed rather than reduced across eight axis-resolved configurations: the baseline at 640, 1024, and 1280 pixels, the two modules alone and together at 1024 pixels, the wavelet module at 1280 pixels, and the transformer. The 1536-pixel rung, the native-resolution encoding at 1024 pixels, and the two mosaic-free configurations are not drawn. Their exchange counts are 210 at 52.1%, 227 at 52.1%, 241 at 53.9%, and 236 at 55.8%, all inside the overall range the figure reports. The mosaic-free run at 1280 pixels nevertheless lies above the 210 to 220 band of the drawn 1280-pixel configurations, so the level of that band depends on the augmentation recipe as well as on input size. Across the drawn configurations, the exchange stays between 210 and 281 pooled confusions and accounts for between 49.3% and 57.3% of all misclassification, while macro-F1 moves 9.86 points, and among them only the resolution ladder reduces it by 71 confusions from 640 to 1280 pixels.

### 3.4. A Detection Transformer and an Oracle Classifier

RT-DETR-l at 1280 pixels gave the highest AP50 of any single model, 57.90 against 53.67 for the best convolutional configuration at the same resolution without test-time augmentation, and the highest detection recall, 96.4%. Macro-F1, however, stood at 62.18 against 64.74.

A crop classifier trained on crops cut from the reference boxes removes detector error entirely. Seven oracle runs across every crop geometry produced macro-F1 values between 54.56 and 57.84 over all 1343 development reference boxes, while two runs of the identical configuration differ by 2.71 macro-F1 with no random seed fixed. Comparing that figure with the detection head requires a common denominator, since the head is scored on the 1244 boxes it matched. Restricted to those same 1244 boxes, which is the population Section 2.7 distinguishes, the oracle reaches 62.70% accuracy and 57.77 macro-F1, against 63.75% and 61.47 for the detection head. That oracle run is the one whose per-crop probabilities were retained, and the highest of the seven on accuracy. It is therefore behind on both, by 1.05 points over an interval of minus 4.32 to 2.25 and by 3.69 points over an interval of minus 7.31 to minus 0.03. The second is at an uncorrected *p* of 0.046 and a corrected *p* of 0.092, and both come from the same image-level paired bootstrap Section 2.9 describes. Neither difference favours the oracle, and neither is large enough to be established under the correction, so what this comparison supports is the absence of a gain rather than the presence of a loss. Read the other way, over all 1343 boxes with every box the detector never found counted as an error, the oracle reaches 62.25% accuracy and 57.50 macro-F1, against 59.05% and 58.98 for the detection head. It is therefore ahead on accuracy by 3.20 points and behind on macro-F1 by 1.48. Supplying the reference boxes therefore does not lift discrimination above what the detection head already reaches on the boxes it finds, so the limit does not lie in the detector’s localisation. The restriction reuses the stored out-of-fold probabilities of the oracle run and involves no retraining and no further model selection.

### 3.5. Effect of Test-Time Augmentation and Ensembling

At 1280 pixels, test-time augmentation raised the plain baseline from 53.28 to 56.56 AP50 and from 92.2% to 95.9% detection recall, whereas macro-F1 did not move: it stood at 64.74 both without augmentation and with it, the difference falling below the third decimal. The gain therefore lies in localisation and not in grading. Class-agnostic detection F1 falls in every configuration tested, by between 4.62 and 6.95 points across the seven pairs of Table 5, so augmentation is an operating point rather than an improvement.

Ensembling used weighted box fusion over the three-member subset named in Table 4, without the transformer. Performance weighting contributes 0.15 macro-F1 over plain fusion. Against the 1024-pixel baseline, the ensemble raises AP50, classification accuracy, and macro-F1 from 51.84, 63.72, and 60.90 to 61.10, 68.98, and 66.22, but most of that margin comes from the resolution and test-time augmentation of its members. Against its strongest member, the baseline at 1280 pixels with test-time augmentation, the gain in macro-F1 is 1.48.

### 3.6. Findings of Evidence-Grounded Attribution

The void reference was first tested as a discriminator in its own right, without any model. Over the twenty-four comparisons of Section 2.8, the area under the ROC curve spans 0.4296 to 0.5609, so the largest departure from chance in either direction is 0.0704 on the merged density column.

That column merges two source labels which describe different appearances, and the reference of Section 2.8 is built on only one of them. The six gated-response descriptors were therefore also scored against each label separately, against the same 810 adequate fillings. Take first the 410 boxes carrying the deficient condensation label, which Table 1 defines by radiolucency at the filling/dentine interface, a generalised reduction in radiopacity, or an interior void under 1 mm. This split is post hoc, as Section 2.8 records. Every one of the six lies above chance there, and the strongest reaches 0.5730, four of them separating at the 1% level under a Bonferroni correction across the twelve comparisons of that split and two under a correction across all thirty-six. On the 77 boxes labelled by a discrete interior lucency, none separates under either correction; every point estimate lies within 0.008 of 0.500, and the smallest uncorrected *p* is 0.82.

The recovery the merged column reports therefore comes from the deficient condensation label and not from the label the operator was designed for. That subgroup of 77 boxes gives a half-width near 0.068, so an effect of up to 6.8 points in either direction remains open there. Whether the two labels differ from each other is not tested. The split is read in Section 4.2. Weak is not the same as absent. Five of the twenty-four are separate from chance at the 1% level after Bonferroni correction across all twenty-four, or three if the twelve comparisons of the source-label split above are treated as part of the same family. The two that fall out are the mean gated response inside the filling and the grey-level spread. The strongest is the eroded interior depth contrast at 0.4296. Its direction is the opposite of the one the descriptor was designed to detect: density defects showing lower depth contrast than adequate fillings, over an interval of 0.3979 to 0.4613. That is consistent with a response dominated by the dark root contour rather than by lucency inside the material. The five that separate are all against adequate fillings, and none is against length defects. The boxes are not independent, moreover, since several belong to the same radiograph, so these intervals are narrower and the corrected *p*-values smaller than a clustered analysis would give.

Attribution was computed with D-RISE at a mask retention of 0.9 under the contrast estimator of Section 2.8, over 180 detections per architecture and identically for a Sobel gradient map and a seeded random map. Because two pressures act in opposite directions across the retention sweep of Table 6, the configuration used throughout sits where 82.1% of masks are informative.

The fall of the uncentred column across the sweep measures the growth of its constant offset and not a loss of information. With the spatial mean removed, the classical map rises across the same sweep and reaches 10.18 at the retention used here, above the contrast estimator’s 9.44. The two forms therefore carry the same information and differ only in the offset they remove and in the per-pixel divisor that Section 2.8 sets out, and neither is preferable on this measure. The choice of retention is carried instead by the match rate, which rises from 16.4% at a retention of 0.5 to 82.1% at 0.9.

Two further checks stand behind that reading: a control in which the detector’s learned parameters were re-initialised, and a convergence check that raised the mask budget to one thousand. The first reports nothing in its own right, and the second leaves the void statistic where it stood.

The void reference was then tested as a reference rather than as a discriminator. If it marks the evidence a model uses for the density axis, a model given that reference as an input channel should attend to it more than one that was not. The native-resolution texture encoding of Section 2.5.3 supplies exactly that channel, so its configuration and the plain baseline were attributed under the same suite at 1024 pixels. Void attribution overlap is 0.9647 for the configuration that receives the channel against 1.0047 for the one that does not, a difference in the opposite direction to the one predicted and not separable at a Mann–Whitney *p* of 0.063, and none of the other five quantities differ. Two cautions attach to this comparison. The two configurations are different models attributed on their own detections, so the two sets of 180 share no box and the test is unpaired. The configuration that receives the channel is also the weaker of the two on the density axis by 3.61 points of F1, so a difference in either direction would have more than one explanation. What the comparison does establish is the absence of the positive evidence the reference would need: giving a model the reference as an input does not raise its attention to that reference. The reference is therefore not validated as a marker of voids, and a null measured against it bounds attention to the reference and not attention to voids.

Table 7 gives the positive control. On the three region-mass and pointing quantities, every Holm-corrected *p*-value against both baselines falls below 0.0001, with D-RISE placing 2.82% of its attribution mass in the box and its ring against 1.49% for the random map. The three region-ratio quantities in the lower half of the table are the ones the nulls are read on, and they are not part of that control.

Against the Sobel edge map, the model-derived map differs on all three region metrics in the convolutional detector: it is 0.061 lower on void attribution overlap at a Holm-corrected *p* of 0.0025, 0.045 higher on apical attribution overlap, and 0.214 lower on the coronal-to-apical ratio. The transformer reproduces the last two, at 0.039 higher and 0.196 lower, and does not separate on void attribution overlap at a Holm-corrected *p* of 0.402. The model-derived map is therefore less crown-shifted and more apically weighted than an edge map, and it lands less of its mass on the void reference than an edge map does. That points to the same explanation as the descriptor result above: a reference dominated by the dark root contour rather than by lucency inside the material.

**Figure 6:**
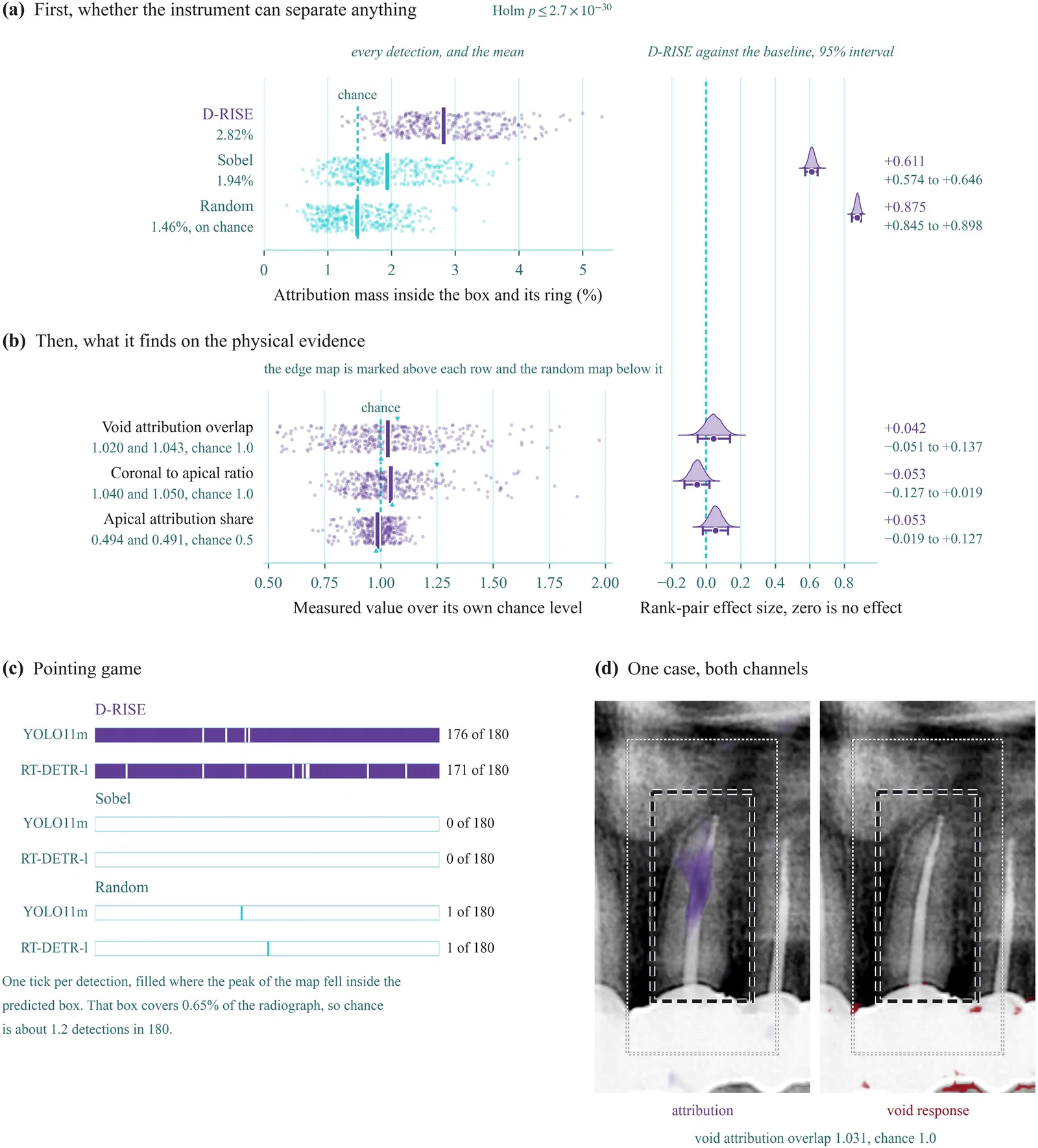
Whether the detector attends to the physical evidence that defines the density criterion, and whether the measurement could have detected it, drawn as one estimation plot. Every panel reads the same 180 attributions per architecture, for the convolutional detector and the set-prediction transformer. D-RISE is the model-derived map, and a Sobel edge map and a smooth random map are the two mandatory baselines, computed on the same detections. On the left of (**a**,**b**) is the raw distribution, one mark per detection, with the mean as a rule and the chance level as a dashed rule. On the right, shared by both, is the rank-pair effect of the model-derived map against a baseline, together with its 95% interval from 10,000 bias-corrected and accelerated bootstrap resamples of the detections. Zero is no effect. (**a**) The share of the attribution mass falling inside the predicted box and its ring reaches 2.82% for the model-derived map, against 1.94% and 1.46% for the edge map and the random map. Those three are pooled over the two architectures, where Table 7 gives the convolutional detector alone at 1.97% and 1.49%, and the random map lands on the geometric chance level of 1.47%. The two effects are 0.611, over an interval of 0.574 to 0.646, and 0.875, over an interval of 0.845 to 0.898, and every Holm-corrected paired Wilcoxon test behind them falls below 0.0001. (**b**) The three quantities that carry an analytic chance level, drawn as value over that level. In the convolutional detector, they measure 1.020, 1.040, and 0.494, against chance levels of 1.0, 1.0, and 0.5. Their effects against the random map are 0.042, over an interval of −0.051 to 0.137, then −0.053, over an interval of −0.127 to 0.019, and 0.053, over an interval of −0.019 to 0.127. All three straddle zero, on the same axis that carries the positive control. (**c**) The pointing game places the peak of the model-derived map inside the predicted box in 176 and 171 of 180 detections, against 0 and 1 for the two baselines, where that box covers 0.65% of the radiograph. (**d**) One correctly graded density defect, with the attribution map on the left and the void-response channel overlap statistic shown on the right. The two do not coincide, and the void attribution overlap is 1.031 against a chance level of 1.0. The heavy dashed rectangle is the predicted box and the dotted one bounds the ring, so the two together are the region of Section 2.8 that the statistic is computed over.

Attribution shows no preference for the void reference over a smooth random map. That map, and not the analytic constant, is the null here. At the three hundred masks used throughout, none of the three region statistics separates from it in either architecture under Holm correction. That correction runs over all twelve comparisons of the suite, which is conservative, because apical overlap and the coronal-to-apical ratio are one quantity in two readings. The excluded effect is bounded at 0.052 for void attribution overlap, 0.008 for apical attribution overlap, and 0.026 for the coronal-to-apical ratio in the convolutional detector. Figure 6b draws the same three comparisons as rank-pair effects, which order the same way and straddle zero on the same axis that carries the positive control. Read against the analytic constant instead, five of the six one-sample intervals exclude it, so the measured values are not literally at chance and are not reported as such. The two architectures are indistinguishable on all six quantities, and attribution does not change when the model is wrong. One comparison has an interval and a corrected test that disagree: the transformer’s void attribution overlap against the random map, whose difference of 0.045 carries an interval of 0.009 to 0.081 that excludes zero at a Holm-corrected *p* of 0.172. Where the two disagree in this family, the corrected test governs, and the difference is not claimed.

### 3.7. Locked Test Set

**Figure 7:**
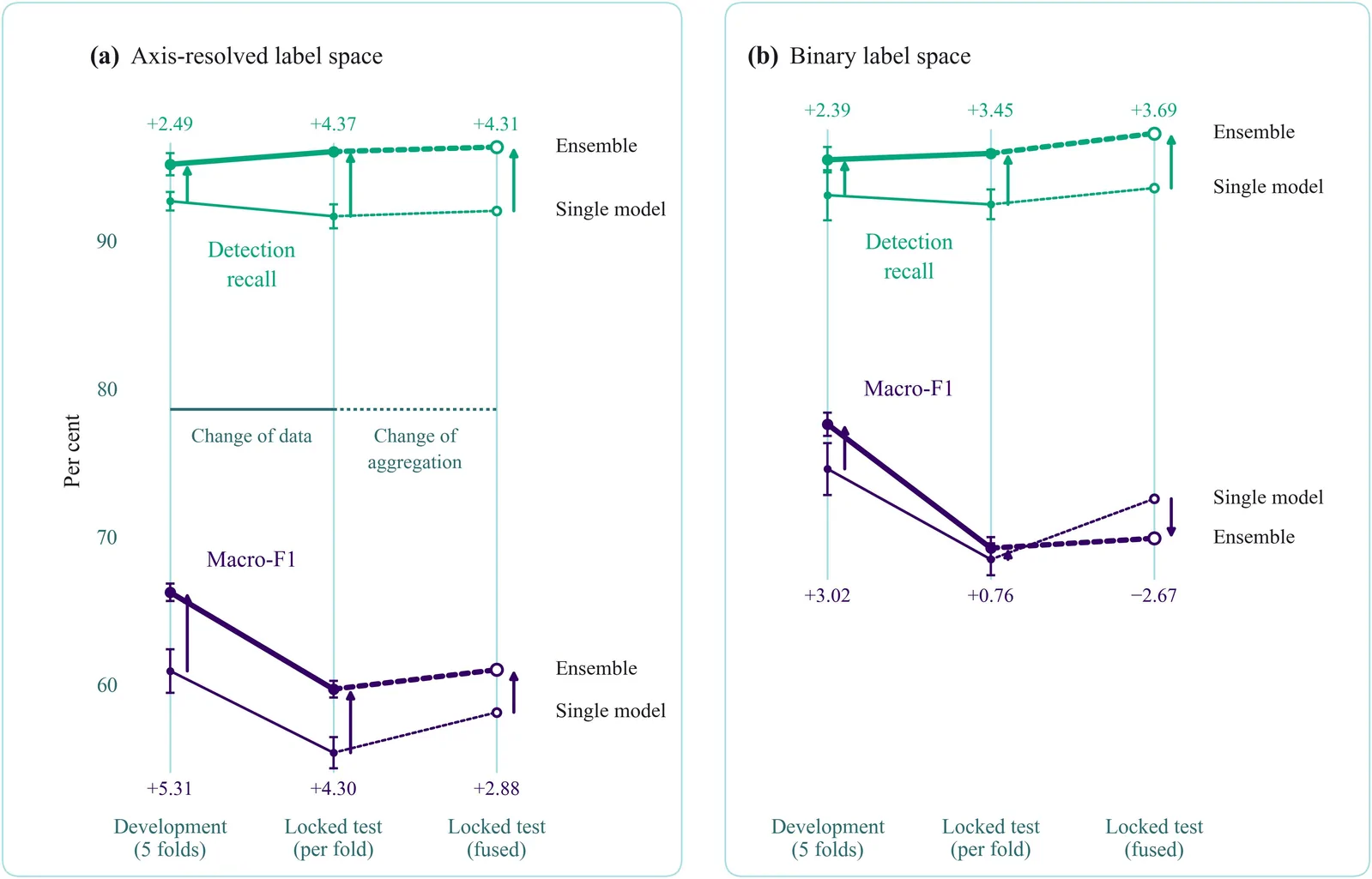
Whether the ensemble gain measured in development transfers to held-out radiographs. Each panel is a slopegraph over three readings of the same two configurations, a single baseline model and the three-member ensemble, which the locked test set chose over a two-member candidate the development folds did not separate from it. The three readings are the five-fold development mean, those same five per-fold models applied singly to the 160 held-out radiographs and averaged, and the five folds fused into the one prediction set a deployed system would use. The first step is drawn solid because it changes the data, and the second dotted because it changes only the aggregation. Colour separates the two measurement axes, green for class-agnostic detection recall and violet for matched-box macro-F1, and line weight separates the two configurations. Both panels sit on one vertical scale, so the two label spaces are compared by position rather than by assertion. The arrow at each reading runs from the single model to the ensemble, so its direction is the sign of the gain and its length the size of it. The three contrasts are printed beside their own band. Error bars are the standard error of the five-fold mean, and the fused reading is one measurement, so it carries an open mark and no interval. (**a**) In the axis-resolved label space, the gain holds its sign on both quantities, falling from 5.31 to 2.88 macro-F1 points across the three readings. (**b**) In the binary label space, detection holds its sign while grading does not. The 3.02 macro-F1 points the development folds predicted fall to 0.76 per-fold on held-out radiographs, and reverse to −2.67 once the folds are fused, which is the reading a clinic would deploy.

The locked test set of 160 radiographs, 19.9% of the working set, was held out before any model was trained, and the cross-validation models are applied unchanged. Figure 7 draws the two label spaces across the three readings reported in Table 8.

Four things follow from the table. First, the three-member ensemble raises AP50 from 45.23 to 56.58 and macro-F1 from 55.38 to 59.68 over a single 1024-pixel baseline model per fold, though most of that margin is the resolution and the augmentation of its members rather than the fusion. Second, against its own best member, the wavelet configuration at 1280 pixels with test-time augmentation, the ensemble is 0.05 macro-F1 points behind per fold and 0.79 points ahead at the fold-fused reading. Third, the held-out set was used to choose between the two candidate ensembles the development folds did not separate, the three-member subset reaching 60.99 macro-F1 against 59.48, so that figure is a selected maximum rather than an unbiased estimate. The development folds had placed the two-member subset nominally ahead, at 66.46 against 66.22 macro-F1, so the held-out set reversed a preference of 0.24 points. Fourth, ten binary readings were measured on the locked test set, four configurations each read singly and fold-fused, and one ensemble read the same two ways. The 72.70% reported here is the highest of them, the others running from 68.94% to 71.97%, so the binary operating characteristics below carry the optimism of a maximum over ten readings, in the same sense as the ensemble figure. Section 3.9 gives all ten, and the reading the development folds would have selected.

The central finding replicates: Detection recall stands at 93.5% against 92.0% at the fold-fused reading, while classification accuracy falls from 72.70% over 304 matched boxes to 60.54% over 299. That is a cost of 12.16 points, with a two-sample binomial interval of 4.69 to 19.63. It is close to the 11.41 points in cross-validation, but resolved far less sharply on a test set of this size. Collapsed onto the binary decision, the held-out axis-resolved predictions give 69.57% against 72.70%, a difference of 3.13 points. The part of the cost that survives the collapse, and is therefore a degradation of the binary decision, is 26% of the held-out cost against 10% in cross-validation. The direction of the cross-validated finding holds: most of the cost is the axis assignment rather than a degradation of the binary decision, but it holds less strongly on held-out radiographs. Every configuration scores lower on the locked test than in cross-validation when read at the same single-model aggregation, with the baseline falling from 51.84 to 45.23 AP50, and the fold-fused rows recover part of that because fusing five models is a different aggregation and not a different measurement. These are the values to read as the performance of the method.

Table 9 gives the operating characteristics of the fold-fused binary decision, which is what Section 4.5 discusses, taking a defective filling as the positive class. Over 304 matched boxes, its confusion matrix is 99 and 45 on the adequate row and 38 and 122 on the defective row.

Under the axis-resolved scheme, the same reading gives a positive predictive value of 46.67% on the density axis, over an interval of 37.41% to 56.16%, and 67.44% on the length axis, over 52.52% to 79.51%. The corresponding sensitivities are 54.44% and 46.03%.

Figure 3c,d draws the held-out confusion structure, which reproduces the cross-validated one. Recalls are 70.5%, 46.0%, and 54.4% for the axis-resolved baseline and 74.5%, 47.0%, and 56.1% for the ensemble, and there are 70 adequate against density confusions against 21 adequate against length, a ratio of 3.3-to-1.

**Figure 8:**
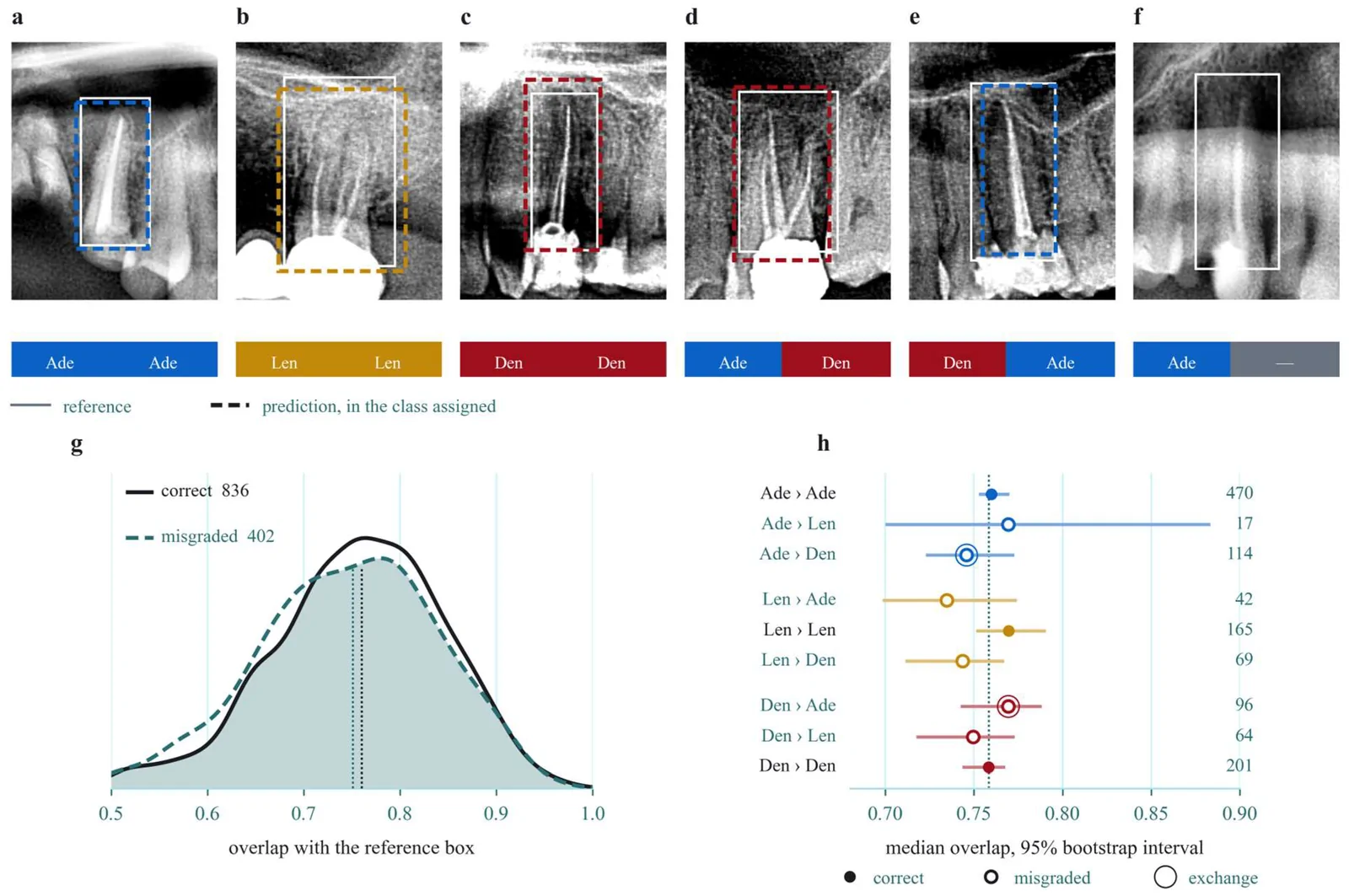
Whether the prediction box lands on the filling does not depend on whether the class attached to it is right. Every reading is the plain baseline at 1280 pixels on the 646 development radiographs, each film predicted by the fold model that never trained on it. (**a**–**f**) Six decisions, one for each of six fates a reference box can have, in the order Figure 3 reads the matrix. The reference outline is thin, solid and white, and the prediction outline is heavier and dashed in the class the model assigned, so a change of colour between the two chips beneath a tile is an error. (**a**–**c**) are the three diagonal cells: an adequate filling, a length defect, and a density defect, each graded as itself. (**d**,**e**) are the two directions of the dominant exchange, an adequate filling read as a density defect and the reverse. (**f**) is a reference box where no prediction reached the 0.25 score floor, which is the one fate that is not a cell of the matrix. Every tile is the same crop, 340 pixels of a 2800-pixel film, windowed on the 2.0 to 99.5 percentile taken inside the root region, so a bright coronal restoration cannot set the upper percentile and hide the material the density axis grades. (**g**) Overlap with the reference box over all 1238 matched boxes, correctly graded against misgraded, as kernel densities with the mass they hold in common filled and each median drawn as a rule. The medians are 0.760 over 836 correctly graded boxes and 0.751 over 402 misgraded ones, 0.009 apart. (**h**) The same overlap by confusion cell, median with a 95% bootstrap interval over 2000 resamples, grouped by reference class, with the vertical dotted line at the pooled median of all 1238 boxes. The nine medians span 0.035, and the two ringed ends of the dominant exchange sit at 0.746 over 114 boxes and 0.769 over 96. Panels (**g**,**h**) count only boxes a prediction reached at the 0.50 intersection-over-union floor of Section 2.7, so the overlap axis is truncated there, and neither panel speaks for the boxes no prediction reached, which (**f**) stands for.

Fusing the five RT-DETR fold models with the settings that improve every convolutional configuration collapses detection recall from 96.3% to 52.9% and AP50 from 52.89 to 42.81, which is why RT-DETR does not enter the ensemble. Figure 8 shows representative detections, including incorrectly assigned axes, and measures the overlap that produced them. Over the 1238 matched boxes of the 1280-pixel configuration, the median overlap with the reference box is 0.760 where the grading is correct and 0.751 where it is wrong, a difference of 0.009, and the 9 cells of the matrix span 0.035. Localisation therefore does not degrade where the grading fails; on the boxes, a prediction is reached.

Augmented inference followed by weighted fusion did not transfer to the binary task. At the fold-fused reading, detection recall rises from 93.54% to 97.23% while macro-F1 falls from 72.54 to 69.87, against a development prediction of a 3.02 macro-F1 gain. The recipe therefore improves the part of the task that was already reliable and leaves the part that was not exactly where it stood.

### 3.8. Sensitivity to the Reporting Threshold

**Figure 9:**
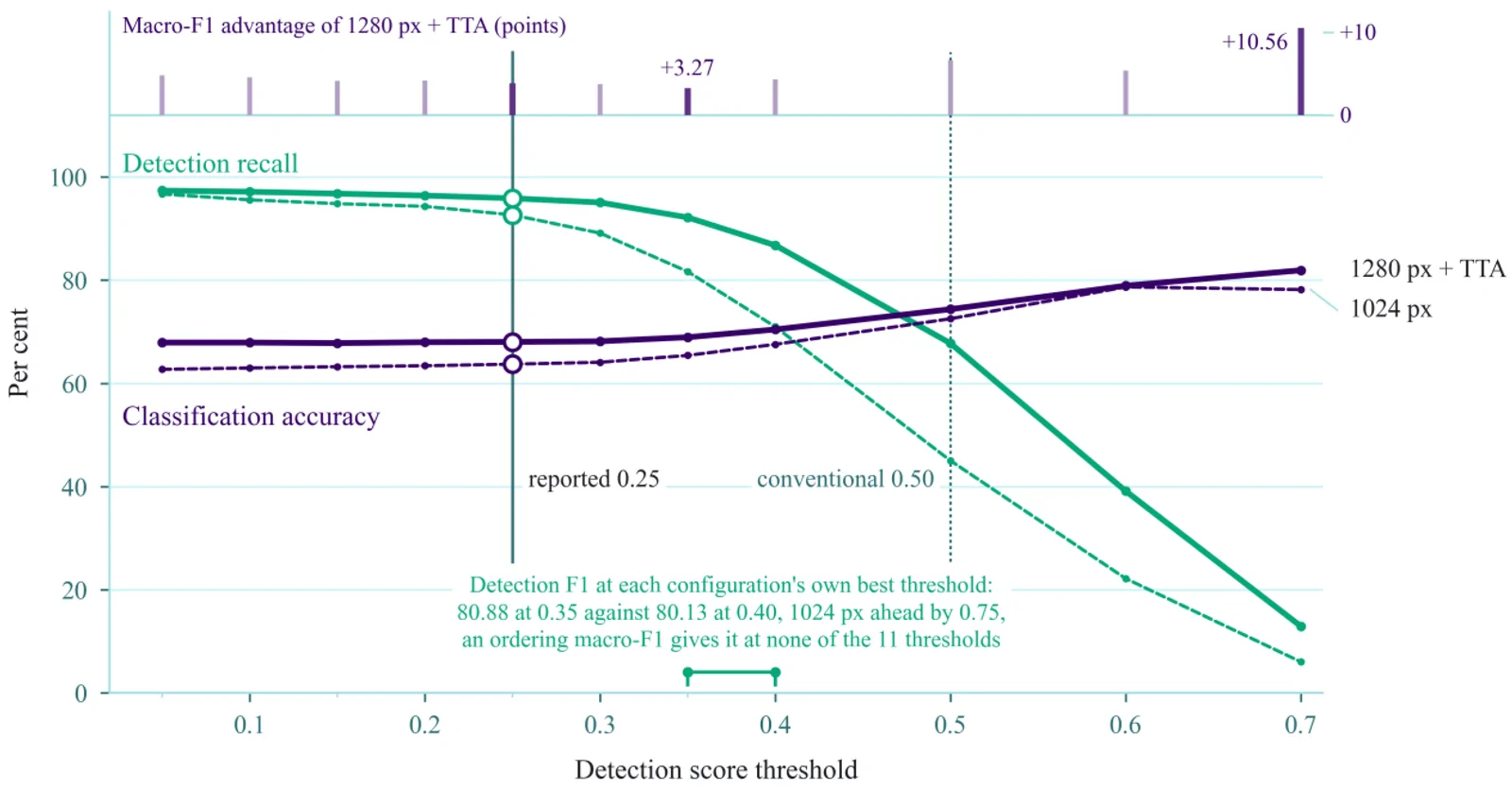
The one free parameter of the separated evaluation, swept. A detection enters the class-agnostic match only if it scores at or above a chosen threshold, and every result in this manuscript is reported at 0.25. The rest of the match is fixed, at an intersection-over-union floor of 0.50 and greedy in descending score. The threshold is swept over eleven values from 0.05 to 0.70 on the development folds for the 1024-pixel baseline and for the 1280-pixel configuration with test-time augmentation. Each point is the mean over the five folds. Class-agnostic detection recall falls strictly at every step of the sweep, from 96.69% to 6.01% for the baseline and from 97.36% to 12.91% for the resolution configuration. The classification accuracy measured over the boxes that survive rises overall, from 62.71% to 78.17% and from 67.91% to 81.91%, though not at every step, with the baseline peaking at 78.68% at a threshold of 0.60. The two curves therefore cross, and no single aggregate can stand for the pair. The solid rule marks the reported threshold and the dotted rule the conventional 0.50, and open rings mark the reported reading on each series. The strip along the top carries the macro-F1 advantage of the resolution configuration over the baseline, with one rung per swept threshold, shortest at 3.27 points at 0.35 and longest at 10.56 points at 0.70. No rung inverts, so within this single-seed run the grading ordering survives any choice of the parameter. Read instead at each configuration’s own best threshold, class-agnostic detection F1 puts the baseline at 80.88 at 0.35 ahead of the resolution configuration at 80.13 at 0.40. That is a lead of 0.75, and it reverses the ordering macro-F1 gives at all eleven thresholds. The two own-best thresholds are bracketed at the foot of the panel. A single aggregate can therefore be moved by a reporting choice that improves nothing.

The score threshold at which detections enter the matched-box comparison affects reporting only, and Figure 9 plots the sweep. At 0.25, the baseline stands at 92.65% recall and 63.72% classification accuracy, whereas 0.50 would have given 45.01% and 72.52%, a rise that reflects the selection of easy detections. The threshold of 0.25 also sits below the detection F1 optimum, which lies at 0.35 for the baseline and at 0.40 for the resolution configuration.

Read at each configuration’s own optimum, class-agnostic detection F1 puts the baseline ahead by 0.75, so a single figure mixing localisation with discrimination can be made to carry either conclusion by a reporting choice that changes no model.

### 3.9. Sensitivity to Labelling Choices and to the Selection of Readings

Of the 71 separated-instrument roots, 61 lie on development radiographs and 10 on the locked test set. Although these roots were treated as background during training, the 1024-pixel axis-resolved baseline placed a box on 56 of the 61 in its out-of-fold predictions, and 78 of its 610 false-positive detections lie on one of them. Ignoring those detections raises detection precision from 67.10% to 70.05% and detection F1 from 77.82 to 79.77, while detection recall is unchanged by construction at 92.63%. The binary baseline moves by a similar amount, from 79.36 to 81.25, so the comparison between the two label spaces is not affected. Detection recall on the reference boxes of the 56 development radiographs that carry such a root is 93.28%, against 92.56% on the other 590, a difference of 0.73 points over an interval of minus 3.94 to 4.78 at a *p* of 0.726. The binary baseline and the 1280-pixel configuration give the same picture, and on the locked test set the fused readings of the two baselines place a box on 9 and 8 of the 10 such roots. Training on these roots as background therefore lowers the measured precision rather than recall, and no suppression of the neighbouring root fillings is detected.

Under the opposite precedence rule, with the model held fixed, the pooled matched-box accuracy of the 1024-pixel axis-resolved baseline, 63.75%, would lie between 60.77% and 66.72%, its macro-F1 between 57.06 and 65.30, and its density F1 between 48.94 and 58.18. The cost of refining the label space would then lie between 8.44 and 14.39 points of matched-box accuracy against the binary 75.16%, so its direction and its order of magnitude do not depend on the precedence rule. The binary decision is unaffected, since both axes are defective under L1. A retrained comparison under the alternative rule would need the 37 roots to be identified, which the annotation does not allow.

On the locked test set, 9 of the 169 defective reference fillings received no matched box at the fused binary reading. Counted as false negatives, they give an end-to-end sensitivity of 72.19%, 122 of 169, over a Wilson interval of 65.00% to 78.39%. This is close to the 71.32% that the product of overall detection recall and matched-box sensitivity gives, and slightly above it, because defective fillings were found at 94.67%, above the overall rate of 93.54%. Table 9 reports the direct count.

The ten binary readings scored on the locked test set span 68.99% to 76.25% in sensitivity, 65.55% to 71.81% in specificity, and 68.94% to 72.70% in matched-box accuracy. The reported reading holds the highest sensitivity and accuracy among them, but not the highest specificity. The reading the development folds would have selected, the fused three-member binary ensemble, gives a sensitivity of 71.52% over 64.20% to 77.85%, a specificity of 68.21% over 60.41% to 75.11%, an accuracy of 69.94% and an end-to-end sensitivity of 69.82%, 118 of 169. Those are the values to read as an estimate free of selection on the test set, and they lie 4.73 points below the reported reading in sensitivity and 0.54 points below it in specificity. For the axis-resolved ensemble, the same rule gives the two-member subset the development folds placed nominally ahead, at 59.48 macro-F1 against the 60.99 reported.

## 4. Discussion

### 4.1. Principal Findings

At 1024 pixels, refining the label space from a binary adequate-versus-defective decision into an axis-resolved one left class-agnostic detection recall at 92.6% against 93.1%. It moved matched-box classification accuracy from 75.13% to 63.72%, so 11.41 points of cost fell on discrimination, over an interval of 8.20 to 14.46. Class-agnostic detection F1 fell by a further 1.54 points, which the multiplicity correction of Section 2.9 does not establish. On the locked test set of 160 radiographs, the figures were 93.5% against 92.0% and 72.70% against 60.54%, a cost of 12.16 points over a two-sample binomial interval of 4.69 to 19.63. Root-filled teeth are found reliably on panoramic radiographs, whereas the axis along which their quality is deficient is not.

### 4.2. Why the Density Axis Resists

Neither an oracle classifier on crops cut from the reference boxes nor four architectural interventions raised the best result available. The physical signature itself is barely there: over the 24 descriptor comparisons of Section 3.6, the area under the ROC curve departs from chance by at most 0.071 on the merged density column. The five that do separate include the strongest, and that one runs opposite to its intended direction. At this resolution, the operator responds to radiolucency, but not selectively to the radiolucency a clinician would call a void.

The arch split of Section 3.1 does not settle this and should not be read as though it did. Missed density defects concentrate in the mandible while false density calls concentrate in the maxilla, which is in part what one threshold applied to two base rates produces. At 1280 pixels, the Youden index, which the base rates do not move, separates the two classes less well in the mandible by 0.194, although the same contrast is not resolved at 1024 pixels and the two resolutions do not differ from each other. If that residue is real, the harder jaw on this axis is the one with fewer density defects rather than the one the projection argument nominates. That is the opposite of what superimposition predicts, but it does not refute it, for a reason no subgroup split can get around: the reference standard is itself a reading of the same projection. If superimposition misleads the annotator, it misleads the label with it, and the contrast then measures the model against that error rather than against the anatomy. The superimposition account is neither supported nor excluded here, and a design that could settle it would need a reference standard from a different modality.

The source/label split is the more informative one. The detector is worst on the label defined in Table 1 by interruptions within the body of the material, but only in one jaw. In the maxilla, it misses 47.6% of those boxes against 16.3% of the deficient condensation label. In the mandible, the two are 44.4% and 45.1%, so the contrast is a maxillary one and not a property of the labels as such. Both jaws are given because reporting only the maxillary pair would make a 31-point gap look like a property of the label pair when it reverses to within a point on the other jaw, on 18 and 102 boxes, respectively. The consequence for the attribution analysis is definitional rather than inferential. Section 2.8 builds its reference on a radiolucent discontinuity inside radiopaque material, whereas Table 1 places 410 of the 487 density defects under a label defined chiefly at the filling/dentine interface or by a diffuse reduction of radiopacity, with interior voids admitted only below 1 mm. For most of those, the reference is therefore named for an appearance the label need not carry. What it responds to instead is measurable, and it is not what the phrase first suggests. Running the operator on synthetic lucencies of known diameter and shape, described in Section 2.8, separates the candidate explanations for that split and, conditional on the split being real, rules two of them out.

Four accounts of that split can be put to the phantom. Position is the first. A lucency straddling the filling/dentine boundary returns a weaker response than the same lucency inside the material, which is the opposite of what a positional account needs. Composition is the second. Taking the 410 as interface lesions alone predicts that the interior-void label should separate the better of the two, and it separates worse. Size is the third, and it divides. At the level of a single lesion, it cannot be tested here at all, because Table 1 records a lucency by its longest dimension while the operator responds to its shortest, and two lesions of the same recorded size can return responses a factor of five apart. At the level of the population, it is consistent with the observation. An interior lucency below 1 mm cannot be labelled non-homogeneous and falls to deficient condensation, and on the phantom such lesions return the strongest responses measured. The account nevertheless rests on a margin of 2.20 points between those responses and the highest a lesion of the 77 can return, smaller than the 2.45 points the operator loses to lesion length alone. It also rests on a mixing fraction the annotation does not record, and on an assumption about what a reader can see on a panoramic film. Every one of these readings is drawn at a 2.24 mm strip, and each inherits a dependence on canal width that this cohort cannot resolve.

A fourth account needs no mechanism at all. If the 77 interior-void boxes carried exactly the effect the 410 shows, a subgroup of that size would fail to reach significance here about nine times in ten, so a pure difference in power could produce the pattern of significance. The intervals weigh against that account without excluding it. For the strongest descriptor, the 77 interior-void boxes give 0.429 to 0.564, an interval that excludes the 0.573 the 410 reach, but by less than one hundredth, without correction and without a test of the difference between the labels. A power account is therefore disfavoured rather than excluded, and that changes the standing of the other three rather than joining them: each of those is an account of a difference between the labels, so each presupposes that the difference is real. The position, then, is this. If the difference between the two labels is real, a positional account is excluded by a measurement that points the wrong way, and an account that treats the 410 as interface lesions alone is excluded by composition. A size account cannot be tested lesion by lesion, and at the level of the population it is consistent with the observation but rests on a margin of 2.20 points, an unmeasured mixing fraction, and an assumption about what a reader can see. If the difference is not real, power accounts for the whole of it and none of the above is needed, although the interval just given makes that the less likely case. This work does not settle which of those two cases obtains, so the split reported in Section 3.6 is not explained here. What the phantom does is narrow the field: it says what a cohort would have to record to settle the question, namely the shape of each void and not only its longest dimension, the width of the canal it sits in, and which deficient condensation boxes carry a sub-millimetre interior void.

What the phantom does establish is narrower and worth stating on its own. The reference is not a void detector in any simple sense: its response is governed by the transverse width of a lucency, it saturates once the structuring element reaches about twice that width, and it is very nearly indifferent to the length that Table 1 actually records. The same 1.04 mm round lucency returns 10.1 at a 9-pixel element, 43.8 at 17, and 47.1 at 31, so even the round-lucency scale is a property of the implementation rather than of root fillings.

The ceiling is not peculiar to this cohort. Against a two-observer cone-beam consensus, the same observers’ consensus reading of the panoramic images scores an F1 of 55.65% for density [18]. That is concordance between two modalities rather than agreement within this one, but it bounds how far panoramic reading can track the state the reference standard is meant to capture, and it does so before any model is involved. A reference standard whose size criterion and whose operator respond to different axes cannot be expected to agree with that operator. That disagreement is a property of the reference standard, and it adds to, rather than replaces, whatever limit the projection imposes. Two caveats attach to all of it. The phantoms carry no root contour, so they cannot test the explanation Section 3.6 offers for the inverted direction of the lucency descriptor, which is attributed there to exactly that contour, and every millimetre here rests on the conversion of Section 2.1, which uses a nominal manufacturer magnification rather than a per-image calibration.

The source/label split reported in Section 3.6 runs the opposite way to the reading the merged column invites: the separation sits on the deficient condensation label, and none is detected on the label the operator is named for. That strengthens the negative result rather than qualifying it. The reference is not a validated marker of voids, and it is on the boxes a void defines that no separation is detected at all. It does not establish two things. It does not establish a zero, because 77 boxes against 810 give a half-width near 0.068. An effect of up to 6.8 points of area under the curve in either direction remains open for that label, which is less than the 7.3 the deficient condensation label itself reaches, and it does not establish that the two labels differ from each other, which would need a test of the difference and is not run here. What is shown is a separation established on one label and not detected on the other.

Agreement on the density criterion is the weakest of the radiographic criteria in the studies available. Undergraduate readers scored against a gold standard set by four endodontists, who themselves agreed at a Fleiss kappa of 0.81, reached kappa values of 0.41 to 0.48 on the length axis and only 0.15 to 0.17 on the density axis, on periapical radiographs [8]. An older assessment of extracted teeth on periapical film, pooling length, density, and taper across uncalibrated examiners, returned a kappa of 0.36, which its authors describe as poor agreement [7]. On panoramic radiographs, formal calibration raised observer agreement for periapical pathology from a range of 0.22 to 0.60 to one of 0.59 to 0.80. In the same study, the objective registrations, among them whether a tooth is root filled at all, were highly reliable in every assessment, including the one before calibration, at intraclass correlations of 0.88 to 0.98 and kappa values of 0.98 to 0.99 [9]. That contrast is the present study’s own division in human form: what a reader must find is reliable, and what a reader must grade is not. None of the three measured inter-observer agreement for filling density among specialists on panoramic radiographs, which is the quantity at issue here, so the density axis is described here by what these studies do show rather than by an agreement statistic on this cohort. A study dedicated to the reliability of intraoral radiographs for endodontic technical quality found the agreement of its two observers to be good. Recordings of the seal nonetheless differed significantly between clinical radiographs and in vitro images of the same extracted roots taken in the same projection, with overlying tissue masking seal defects [37], so the imaging conditions contribute to the variation as well as the reader. Calibration is therefore a lever on this modality, even though its effect on filling density has not been measured.

The model’s own errors fall on the same axis. The adequate-against-density exchange carries 244 errors in cross-validation against 79 for adequate-against-length, a ratio of 3.1-to-1, and that ratio reappears at 3.3-to-1 on the locked test. The two observations are consistent with one another, but the second does not establish the first, because a model trained on labels of a given reliability reproduces their structure whatever the cause of that structure is. Section 4.4 records label noise as an alternative this study cannot exclude.

### 4.3. What the Attribution Analysis Shows and Does Not Show

A null result is interpretable only if the instrument can detect something. The global maximum of the D-RISE map falls inside the target box in 97.8% and 95.0% of detections against 0.0% and 0.6% for a Sobel gradient map and a seeded random map, at *p* below 0.0001.

The remaining results are bounded non-detections rather than failures to measure. Against a smooth random map, none of the three region statistics separate at the mask budget used throughout, two of the three being one quantity in two readings, and the excluded effect is bounded at 0.052, 0.008, and 0.026 in the convolutional detector. Raising that budget separates two of the three: relative to a random map, the attribution is shifted apically and away from the coronal restoration. The suite therefore does resolve regional structure at this scale when given enough masks. What it does not resolve at either budget is any preference for the void reference, the bound on which is 0.058 at the higher budget. Evidence below the scale of the occlusion regions would not register, and the criterion sits at or below that scale, so no conclusion is drawn here about a localised substrate at the scale of the criterion.

The void reference is not validated as a marker of voids, and this is the limit that matters most for what the attribution analysis can be asked to show. The descriptor search in Section 3.6 places its largest departure from chance on the merged density column at 0.0704 and reverses the sign of its strongest descriptor. A model given that reference as an input channel, moreover, does not attend to it more than one that was not. The lesson is transferable and is the one this study would put forward in place of the claim it set out to make. An annotation-free reference derived from the physical definition of a criterion is attractive because it needs no expert opinion, but it must itself be shown to mark the evidence before a null against it carries meaning. The test for that is cheap, since a model can simply be given the reference as an input and its attribution measured.

One limit of the attribution analysis follows from the label definitions rather than from the attribution method. The void reference is defined on a discontinuity inside the material, and Table 1 separates the two density source labels by location. For the 410 deficient condensation boxes, which Table 1 defines chiefly at the filling/dentine interface or by a diffuse loss of radiopacity, the reference is therefore measuring something the label need not turn on.

Whether the scale of the structuring element governs these descriptors was checked rather than assumed, by rerunning the response at 17, 31, and 41 pixels. Each statistic keeps its side of chance across an element that more than doubles, so the element scale does not govern the statistics it recomputed, and it leaves the inversion of the lucency descriptor untested.

Two elements of the protocol transfer. Localisation and discrimination belong apart wherever an object is easy to find, and its grade is hard to assign. Occlusion-based attribution on radiographs also needs a high retention rate, since the retention of 0.5 used for natural images [33,34] leaves only 16.4% of masks informative on this material. At a high retention, moreover, the classical weighted sum must be read against its near-constant offset, as the contrast of Equation (5) does, which is a correction for the regime rather than a different estimand.

### 4.4. Comparison with Previous Studies, and Limitations

Table 10 places this study beside the closest published work. Çelik et al. segmented the filling on periapical radiographs and assigned a length category from the segmented tip to a hand-marked apex [20]. Ayhan et al. trained the detector used here, YOLO11m, on periapical radiographs of extracted teeth, merging both directions of the length axis [17]. Jin et al. developed YOLOv5 on periapical radiographs from one centre and tested on 500 from a second, folding length and density into one binary decision [16]. None of them read one annotation at the two levels of the clinical rubric, acceptable or not first and only then deficient along an axis, nor did the two commercial-platform studies score panoramic features as independent binary decisions [18,19].

The two platform studies bear on Section 4.2 and were obtained independently. On 55 panoramic radiographs scored against two observers, the platform reached F1 values of 14.29% for voids and 8.33% for short fillings, with accuracies of 55.81% for obturation adequacy and 62.79% for density adequacy [19]. The quoted metric differs between the features; the F1 on those last two stands at 62.75% and 75.00%. Those accuracies rest on specificities of 30.77% and 16.67%, so on both of them the platform called almost every filling adequate. Its successor, scored against a two-observer cone beam computed tomography consensus, reported adequate density at an F1 of 42.67% with a 95% interval of 27.69% to 56.31% [18]. The observers’ consensus reading of the same panoramic radiographs reached 55.65%, and the platform exceeded 94% accuracy on all five features on cone beam computed tomography.

Choi et al. on 1638 panoramic radiographs [11] and Kazimierczak et al. on 4148 teeth [38] located root-filled teeth at a similar level. Khurshid et al. on 2235 radiographs from three countries reported a best mAP50 of 82.0% from Faster R-CNN, as a mean over six restoration classes, of which the root canal-treated tooth is one [12]. Finding a root-filled tooth is therefore reliable across cohorts and countries. Deciding what is wrong with the filling is not.

Three properties of the last row are absent from most of the others. Localisation and discrimination were measured separately, where every other quality-grading study reported one figure carrying both error sources and neither platform study reports grading conditioned on matched teeth [18,19]. Measuring them apart is not itself new outside dentistry, where a consortium framework guides the problem-aware selection of validation metrics [39] and diagnostic tools decompose detection error into localisation error, confusion with similar objects, and confusion with background [40,41]. What the last row adds is that decomposition is applied to a clinical grading task read at two nested levels, and the scope of the claim is the dental grading literature rather than detection at large. The test set was locked before training. A review of thirteen studies identifies patient selection and the absence of external validation as the dominant sources of bias in this literature [15], and this study also lacks the second. Every headline comparison carries an interval, which appeared in only two prior rows, both for a commercial platform the authors had not trained [18,38].

Several studies hold advantages this study does not. Jin et al. validated externally at a second centre [16], and Kazimierczak et al. used a cone beam computed tomography anchor [18]. On the reference standard, however, the position is level rather than behind. Both panoramic quality studies used the consensus of two independent observers [18,19], and the annotation here was read independently by two specialist endodontists and adjudicated by a third. Two other studies used three readers: Khurshid et al., who cross-reviewed 15% of their images and reported a Cohen’s kappa of 0.89 [12], and Dennis et al. [42]. What this study offers is scale: 806 panoramic radiographs and 1668 graded roots against 189 root-filled teeth in the panoramic quality study that reports that count [18].

Against that standing, seven limits bound what the study supports. The phantom work of Section 4.2 is the first. Its lucencies are uniform ellipses of a single intensity on a uniform strip varied only in width, with no root contour, no trabecular background, and no projection blur, so they characterise the operator on those scenes rather than on radiographs. The response of the operator depends on the width of the strip a lucency sits in, and the cohort records no canal width, so where this cohort falls on that dependence is not established.

The second is the cohort itself. The radiographs come from a single centre, and no external cohort was used. They also come from one panoramic unit, and examinations of poor quality were excluded before analysis, so performance on unselected routine examinations is not established. The tooth number is unrecorded, and the filling material, sealer, and obturation technique are those of the institutional protocol stated in Section 2.1 rather than a record kept root by root. Each bounds a different claim. Without a tooth number, no canal width can be recovered, even approximately. A root filled outside that protocol would be read against the wrong radiopacity comparator in Table 1. Moreover, the technique sets how often a void occurs and where, and therefore sets the very ratio of the two labels whose difference Section 4.2 is about, so that ratio may not transfer to other techniques. The working set of 806 radiographs and 1668 boxes is a modest cohort for a three-class problem.

The third is the label space. It omits overfilling, so the length axis runs in one direction only. Taper was not annotated either, since it is an objective of canal preparation rather than a requirement for the filling itself [1], although the observer studies discussed in Section 4.2 do score it beside length and density [7,8]. The rubric applied here therefore sits between two poles, the single-level criterion of the Belgian panoramic survey [43] and the three criteria those observer studies score. Coronal restoration quality is not annotated, although it is associated with periapical health [2,4,44] and, jointly with filling quality, in a further cohort [3]. Single-label annotation, moreover, cannot express a tooth deficient on two axes at once.

The fourth is label noise this study introduced. Of the 95 separated-instrument boxes dropped before training, 24 were the only annotation their radiograph carried, so those films left the working set, which is what reduces 830 examinations to 806. The other 71 sit on 66 radiographs that carry other annotations and were retained, so those roots stay in the images with no label of their own, and the detector is trained to read a real root filling as background. It could act against detection recall on exactly the teeth a clinician would want flagged. The 71 boxes remain in the source annotation, and Section 3.9 scores the detector against them. The detector places a box on most of these roots despite that training signal, so the cost falls on measured detection precision rather than on recall, and no reduction in recall is detected on the films that carry them.

The fifth is the projection. Panoramic radiography is not the projection on which endodontic treatment is usually assessed, which is intraoral periapical radiography [45], so the labels were assigned on a projection a clinician would not choose. It also means that the millimetre thresholds of Table 1 are operational radiographic criteria, read through magnification, distortion, and superimposition, rather than exact anatomical measurements. The evidence on whether the projection matters is not unanimous. A comparison of 138 endodontically-treated teeth imaged all three ways found a significant difference in obturation quality between each of the two projections and cone beam computed tomography, but none between the two projections themselves. Panoramic and periapical radiographs agreed on lesion identification at a kappa of 0.956 [46]. A second property of the material bounds the reference standard further. An exploratory clustering of the radiographs on acquisition radiometry, part of the unplanned examination described in Section 2.10, separates groups that differ in class prevalence at a chi-square *p* of 1.89 × 10^−5^, so a device signature is associated with the labels in this single-centre material. Nothing in the reported results exploits it, but it is the kind of association a model trained and tested within one centre can learn in place of the pathology.

The sixth is that every value rests on a single draw. Every configuration scored lower on the locked test set than in cross-validation on average precision, when both are read as means over single-fold models, with the axis-resolved baseline falling from 51.84 to 45.23 AP50 and the length class furthest in recall, 46.0% against 56.6%. The fold-fused readings of Table 8 recover part of that, because fusing five models is a different aggregation and not a different measurement. YOLO11l at 1280 pixels completed three of the five folds, and the framework accepts but does not implement augmented inference for RT-DETR, so the transformer could not enter that comparison. A second seed moved the five-fold mean macro-F1 from 64.74 to 62.10, and the five-fold mean classification accuracy from 67.49% to 63.47%, below the 63.72% of the 1024-pixel baseline, so the ordering across the resolution ladder is not stable to it, and 1280 pixels is not an established optimum. No a priori power or sample size calculation was performed, and the cohort was assembled from examinations already carrying a root filling, so it is enriched relative to an unselected screening stream and the predictive values in Section 4.5 will differ there even if sensitivity and specificity carry over.

The seventh is the central inference itself. The claim that the density axis is bounded by the evidence in the projection rests on convergent negative results. The measurement that would make it direct is an agreement statistic on this cohort, and although every radiograph was read twice, the two readings were not kept apart, so that statistic cannot be recovered from the annotation as it stands. This is the limitation that bears most directly on the grading results, because label noise lowers any measured agreement between model and reference, and its size on this cohort is unknown. Label noise on the density axis therefore remains an alternative this study cannot exclude. Some formulations train with annotator disagreement rather than around it, among them the regularised estimation of an annotator confusion matrix [47] and the wider set of remedies surveyed for label noise in medical image analysis [48]. These were not attempted here and are the most direct response to that alternative.

What was not tested bounds the conclusion as much as what was. Every configuration here is a supervised detector of one generation initialised from COCO-pretrained weights, and four branches were not opened. The first is self-supervised or foundation-model pretraining on dental radiographs. The second is a segmentation-first pipeline that would measure the apical termination against a delineated apex instead of inferring it from a box. The third is a hierarchical or multi-label formulation matching the staged rubric the Introduction describes. The fourth is a cost-sensitive or pairwise-margin loss aimed at the adequate against density exchange. No search over learning rate, loss weighting, class sampling, or augmentation was performed either, and since the mosaic result of Section 3.2 shows that the augmentation recipe alone moves macro-F1 by more than any module tested, the recipe rather than the architecture may be where the remaining room is. Two estimators of irreducible error that a singly annotated set admits were also left unused: a confident-learning estimate over the stored out-of-fold predicted probabilities and the disagreement between the five per-fold models and the second seed.

Re-reading this cohort with the per-reader labels retained would yield the agreement statistic the present annotation cannot supply, converting the central inference into direct evidence and placing the model against a human upper bound. Repeating the analysis on periapical radiography would test the criterion where it is assessed clinically.

### 4.5. Clinical Implications

One implication follows from Section 4.2 rather than from the detection figures. The reference standard records a void by its longest dimension while the operator responds to its shortest, so two lesions that Table 1 calls the same size can return 8.8 and 46.7. A density flag is therefore not a statement about how large a void is, and a clinician should not read the absence of one as evidence that a void is small. What the flag tracks is transverse width, the dimension Table 1 does not record. A root canal runs roughly vertically in the image, so a void’s transverse dimension lies along the horizontal axis, and that is the axis along which a moving-slit panoramic system’s magnification varies with the patient’s position relative to the image layer. The dimension the reference standard ignores and the dimension the projection degrades most are therefore the same dimension.

On the locked test set, the fused binary decision, the highest of the ten binary readings scored there, found 93.5% of the root fillings and graded 72.70% of matched boxes correctly, the unit throughout being a treated root and not a tooth. Taking a defective filling as the positive class, sensitivity was 76.25%, specificity 68.75%, the positive predictive value 73.05%, and the negative predictive value 72.26%. In counts, 38 of 160 defective fillings were returned as adequate, and 45 of 144 adequate fillings were flagged. Counting the 9 defective fillings the detector never found as false negatives, end-to-end sensitivity for a defective filling is 72.19%, and the reading the development folds would have selected gives 69.82%. At those figures, the decision could serve as a triage aid within the setting studied. It may be useful for teaching and for flagging teeth that require a periapical view. In an audit, it is useful only where each flagged tooth is confirmed by a reader rather than counted directly, since at this sensitivity and specificity an uncorrected count would overstate a true prevalence of 20% as 40%. It is not a screening instrument and not a prevalence estimator. The margin of 20.07 points over the majority class is in any case a weak reference, since calling every filling defective is not a clinical alternative. What such a margin does not supply is the net benefit of acting on the decision across the range of threshold probabilities a clinician might hold, which is what a decision curve reports [49] and which this study does not compute. Both criteria it merges are associated with apical periodontitis in independent cross-sectional cohorts, inadequate length and non-homogeneous filling alike [2,3,4,5].

The two errors do not carry the same cost. A filling wrongly called adequate leaves a tooth that would require a periapical view unexamined, which is the error the use cases in Section 1 are least able to absorb. A filling wrongly called defective, by contrast, costs a second view and the small exposure it carries. At the operating point reported here, the first error occurs in 23.75% of matched defective fillings, and in 27.81% once the fillings the detector never found are counted, whereas the second occurs in 31.25% of adequate ones. Trading one against the other would need a decision threshold on a calibrated score, which this study does not provide, and not the score threshold of Section 2.7, which is a detection floor and moves recall as well as grading.

The axis-resolved reading is not deployable. It grades 60.54% of matched boxes correctly against a majority class of 48.83%, a margin of 11.71 points. It returns a positive predictive value of 46.67% on the density axis, so fewer than half of the density flags it raises are correct.

The cost of running the configuration these figures describe is small. Every clinical quantity above comes from the binary decision at 1024 pixels read with the five per-fold models fused, which was timed separately at 9.8 milliseconds per radiograph for one fold model on the card used here, so 49.1 for the five. On a processor alone, with no graphics card, the same single pass takes about 281 milliseconds and the fused reading about 1.4 s, a figure that bounds the order of magnitude and not the value. Both are well inside what a chairside or an audit workflow would absorb, and the second matters more than the first, because a dental clinic is far likelier to hold the processor than the card. The ensemble of Section 3.5 is the expensive object, at 354 milliseconds for its fold-fused reading against 12.4 for a single pass, and it is not the system characterised here. None of that bears on why the density axis resists, since inference cost and the ceiling on accuracy are different quantities.

The binary decision could suit teaching and reader-confirmed audit once externally validated, but not the indication of retreatment, and the axis along which a defective filling departs should not be reported.

The system cannot report material extruded beyond the apex, which this label space does not carry. The prevalence literature associates extrusion with apical periodontitis more strongly than a short filling, at odds ratios of 2.51 against 1.76 in the largest of these cohorts [2]. Every comparable study cited here gives extruded material a place, as a separate variable, as a third length category, or folded into an unacceptable length, so its absence here is a difference from the field and not a convention of it. A systematic review of primary root canal treatment outcomes identified four conditions that significantly improved the outcome, among them a root filling extending to within 2 mm of the radiographic apex and a root filling with no voids [50]. An extruded filling fails the first of those two. Whether it also carries voids is a separate question that extrusion does not settle, and this study does not measure. As Section 2.1 records, such roots were labelled on the position of the main filling mass, so most of them fall into the adequate class and are counted as adequate wherever an adequate class appears, including the specificity of Table 9. A system trained on this scheme therefore cannot flag extrusion and will call most extruded roots acceptable.

Radiographic obturation quality is, moreover, one component of endodontic treatment quality and not a measure of it. A filling records the end of a sequence whose earlier steps the radiograph does not show. How well the canal was cleaned and disinfected depends on the irrigation protocol, which governs the removal of debris and smear layer and how far the irrigant penetrates [51]. Access cavity design in turn influences canal location, instrumentation, irrigation, and the obturation that follows [52]. A radiographic grade of the filling is therefore not a surrogate for the quality of the treatment as a whole, and the decisions discussed above concern the filling alone.

Every figure above also belongs to the setting in which it was measured: one centre, one panoramic unit, fillings placed by cold lateral condensation with an epoxy resin sealer under one institutional protocol, and examinations that had already passed exclusion for image quality, which removed 212 of the 1129 screened. In routine use, a system of this kind would encounter blurred, distorted, or heavily restored films, and other devices, sealers, and techniques, so its sensitivity and specificity describe this setting and not panoramic radiography in general. External validation on other centres and devices, and on unselected examinations, is required before transfer can be claimed.

## 5. Conclusions

This study read root canal filling quality at the two levels of the clinical rubric. A binary adequate-versus-defective decision was refined into an axis-resolved one that separates length from density, and localisation and discrimination were reported as separate quantities. Refining the label space left class-agnostic detection recall where it was and took its cost out of discrimination, both in cross-validation and again on a locked test set. Root-filled teeth are found reliably on a panoramic radiograph. The axis along which their quality is deficient is not recovered at a level that would support reporting it.

That boundary is not moved by the models applied to the radiographs. At 1024 pixels, four architectural interventions left the limit where they found it. Their intervals put the smallest detectable density-axis difference at 8.2 to 10.8 points, so what is established is the absence of an improvement larger than that, not the absence of an effect. Two of the four were retested at 1280 pixels, the highest rung of the resolution ordering. The wavelet module moved neither macro-F1 nor the density axis by a resolved amount, and the void channel resolved a loss of 9.05 points on the density axis, over an interval of minus 14.03 to minus 4.00, against a gain of 2.07 in class-agnostic detection F1 over 0.73 to 3.35. That is a trade-off between localisation and grading rather than an improvement.

The signature of a discrete radiolucent discontinuity inside radiopaque material could not be recovered at usable strength from the panoramic pixels. A minority of the descriptor comparisons do separate from chance, so that signal is weak rather than absent. It is also the defining signature of only 77 of the 487 density defects. Table 1 defines the other 410 chiefly by the filling/dentine interface or a diffuse reduction of radiopacity, admitting interior voids only below 1 mm. Scoring the two source labels apart, in a post hoc split, finds the weak separation on those 410 and none on the 77. The operator therefore shows no detected separation on the boxes a void defines. For the strongest descriptor, the interval on those 77 boxes excludes the effect the larger label reaches, though by less than one hundredth, which makes it a stronger statement of the same negative rather than a weaker one. It is bounded all the same, both by the 77 boxes available to make it and by the fact that the two labels were never tested against each other. The annotation-free reference built from that signature is not itself validated as a marker of voids, because a model given it as an input channel does not attend to it more than one that was not. What the attribution analysis bounds is therefore attention to the reference, not attention to voids.

The levers still open are levers on the data. Rebuilding the learning curve on unchanged validation partitions moved the density axis by more than the whole usable resolution range delivered, and nothing in that range establishes where it stops. The augmentation recipe matters as well: turning mosaic augmentation off at 1280 pixels costs 4.24 points of macro-F1, more than any architectural change moved it. None of the architectural or optical interventions tested here raised the limit. They were confined to two lightweight modules, an input encoding, input resolution, and detector family, and the input encoding in fact lowered it at 1280 pixels. The limit is in part a property of the sample. What remains cannot be separated from label noise on the density axis by an agreement statistic, which this cohort’s annotation record cannot supply, and other estimators of irreducible error were not applied here.

A panoramic system of this kind should report whether a root filling is acceptable or defective. Conditional on the root filling having been found, and at the highest of the ten binary readings scored on the locked test set, it does so at a sensitivity of 76.25% and a specificity of 68.75%, and the reading the development folds would have selected gives 71.52% and 68.21%. Counting the defective fillings the detector never found as false negatives, end-to-end sensitivity is 72.19%. These figures come from one centre, one panoramic unit, and one obturation protocol, on examinations that passed exclusion for image quality, and they suggest a potential role in teaching and reader-confirmed audit, not in screening, that external validation has yet to establish. What the system should not do is report along which axis the filling departs. Used that way, it grades every root filling in an image already acquired and hands the axis-resolved judgement to the periapical radiograph. It cannot report extrusion beyond the apex, which this label space does not carry, so the classification does not capture every form of technically inadequate root canal filling.

## Figures and Tables

**Table 1:** The five source labels and the radiographic criterion applied to each. The millimetre thresholds are operational criteria read on the panoramic image, not anatomical measurements.

Label	Radiographic Criterion Applied	Boxes
Adequate Filling	Terminates 0 to 2 mm short of the radiographic apex. Exhibits uniform radiopacity (distinctly greater than adjacent dentine) with no voids within the material or at the filling/dentine interface.	810
Deficient Condensation	Presence of radiolucency at the filling/dentine interface, or a generalised reduction in radiopacity. Includes internal voids measuring < 1 mm.	410
Short Filling	Terminates > 2 mm short of the radiographic apex.	371
Separated Instrument	Presence of a discrete radiopaque object with instrument morphology within or beyond the canal. This classification supersedes other concurrent defects.	95
Non-homogeneous Filling	Contains one or more discrete radiolucent interruptions strictly within the material body, measuring ≥ 1 mm in their longest dimension.	77

**Table 2:** The source annotation grouped by axis, and the two derived label spaces. Imbalance is the ratio of the largest to the smallest class in each row.

Scheme	Classes	Boxes Per Class	Imbalance
Source annotation, grouped by axis	Adequate, Length, Density (410 deficient condensation and 77 non-homogeneous), Separated instrument	810/371/487/95	8.53 to 1
L1 binary	Adequate, Defective	810/858	1.06 to 1
L2 axis-resolved	Adequate, Length, Density	810/371/487	2.18 to 1

**Table 3:** Detection models, their static cost as reported by the framework for the three-class configuration at its 640-pixel profiling reference, and the time each takes on one radiograph. Each time is the median of five passes over the 150 timed radiographs of the locked test set. The passes were taken in a different random order each time, so that any drift on the card cannot align with the order of the table, and ten warm-up images were discarded, and the device was synchronised after every image. The figure covers preprocessing, the forward pass and, for the convolutional detectors, non-maximum suppression, which the set-prediction transformer does not use. It excludes a residual of 13.2 to 14.3 milliseconds per image that separates these times from the wall clock. That residual covers reading the file, dispatching the call, and synchronising the device, none of which was timed separately. The largest disagreement between passes on any one configuration was 1.57 milliseconds, which bounds what this measurement can resolve.

Model	Parameters	GFLOPs	Input Sizes Used	Time Per Radiograph (ms)
YOLO11m	20,055,321	68.2	640, 1024, 1280, 1536	7.3, 10.8, 12.4, 15.2
YOLO11m with RASA	20,363,039	69.2	1024	10.5
YOLO11m with WVB	21,646,556	69.3	1024, 1280, 1536	11.0, 12.7, 16.1
YOLO11m with RASA and WVB	21,954,274	70.3	1024	11.0
YOLO11l	25,312,793	87.3	1280	13.8
RT-DETR-l	32,812,241	108.0	1024, 1280	20.5, 29.3

**Table 4:** Cross-validated performance on the 646 development radiographs, as five-fold means except H9, which completed three folds and is not directly comparable. AP50 is computed at a confidence of 0.001 and the detection and classification metrics at a score threshold of 0.25. Graded correctly is the joint quantity: the share of reference boxes that were both found and then graded correctly. It is the product of detection recall and classification accuracy. All rows except T1 use the axis-resolved label space L2. Stage T3 covers the resolution rungs at 640 and 1280 pixels, which the input column distinguishes, and T3 + TTA, H7 + TTA, and H2 + TTA are the members of the ensemble.

Stage	Configuration	Input (px)	AP50	Detection Recall (%)	Classification Accuracy (%)	Macro-F1	Graded Correctly (%)
T1	L1 binary baseline	1024	64.88	93.1	75.13	74.56	69.92
T2	L2 baseline	1024	51.84	92.6	63.72	60.90	59.03
T3	L2 baseline	640	45.04	90.8	58.84	54.88	53.43
T3	L2 baseline	1280	53.28	92.2	67.49	64.74	62.23
T4	L2 baseline	1536	54.86	91.8	67.28	64.05	61.75
A0	L2 baseline, mosaic augmentation off	1280	51.99	92.4	63.95	60.71	59.11
A1	L2 baseline, mosaic off, rectangular input	1856	53.48	90.8	65.28	62.27	59.25
H1	RASA	1024	51.58	91.1	64.30	61.36	58.60
H2	WVB	1024	53.58	94.0	65.76	63.56	61.81
H4	RASA and WVB	1024	52.65	90.2	64.28	61.35	58.00
H7	WVB	1280	53.67	92.7	66.36	63.75	61.55
N1	NRT input encoding	1024	51.92	92.9	65.00	61.04	60.36
N2	NRT input encoding	1280	52.00	92.2	66.44	61.71	61.25
D2	RT-DETR-l	1280	57.90	96.4	66.44	62.18	64.04
T3 + TTA	L2 baseline, test-time augmentation	1280	56.56	95.9	68.03	64.74	65.22
H7 + TTA	WVB, test-time augmentation	1280	56.04	95.8	67.03	64.25	64.22
H2 + TTA	WVB, test-time augmentation	1024	55.81	96.2	65.53	62.65	63.06
H9	YOLO11l, test-time augmentation (three folds only)	1280	56.84	96.2	66.37	61.74	63.85
Ensemble	T3 + H7 + H2, performance weighted	—	61.10	95.1	68.98	66.22	65.63

**Table 5:** Effect of test-time augmentation on class-agnostic detection, all five-fold means. T5 is the binary baseline at 1280 pixels, and H10 is the binary scheme with the wavelet module at 1024 pixels.

Configuration	Recall (%)	Recall with TTA (%)	Detection F1	Detection F1 with TTA	Change
T2 baseline, 1024	92.65	96.01	77.99	71.03	−6.95
T3 baseline, 1280	92.21	95.87	76.91	71.22	−5.68
H2 wavelet, 1024	93.99	96.23	77.73	71.03	−6.70
H7 wavelet, 1280	92.75	95.81	76.76	70.05	−6.71
T1 binary, 1024	93.05	95.78	79.39	73.94	−5.45
T5 binary, 1280	94.29	95.94	80.37	74.14	−6.23
H10 binary wavelet, 1024	93.04	96.47	78.79	74.17	−4.62

**Table 6:** Occlusion-rate probe on the first cross-validation fold over 30 stratified target detections with 24 masks per target at each rate. Dynamic range is the peak-to-trough spread normalised by the mean absolute value. The classical estimator is given both as the raw weighted sum and with its spatial mean removed, which is its closest counterpart to the contrast estimator.

Retention	Masks Matching the Target (%)	Targets with No Measurement	Classical, Uncentred	Classical, Mean Removed	Contrast Estimator
0.30	11.9	10 of 30	3.24	5.44	8.84
0.50	16.4	3 of 30	1.97	5.25	7.93
0.70	36.0	1 of 30	1.31	6.81	9.45
0.80	56.9	0 of 30	0.94	8.70	10.91
0.90	82.1	0 of 30	0.51	10.18	9.44
0.95	92.5	0 of 30	0.30	10.50	7.54

**Table 7:** Attribution against the two mandatory baselines, all six quantities. Values are for YOLO11m unless stated otherwise. The chance level is 1.0 for void attribution overlap and the coronal-to-apical ratio, 0.5 for apical attribution overlap, and 0.0147 for mass inside box and ring, which is the area share of that region.

Quantity	D-RISE	Sobel	Random
Mass inside box and ring	0.0282	0.0197	0.0149
Energy pointing game	0.0178	0.0094	0.0066
Pointing game, YOLO11m	0.978	0.000	0.006
Pointing game, RT-DETR-l	0.950	0.000	0.006
Void attribution overlap	1.020	1.082	1.003
Apical attribution overlap	0.494	0.448	0.490
Coronal-to-apical ratio	1.040	1.254	1.049

**Table 8:** Performance on the locked test set of 160 radiographs. Thirteen configurations were scored on it, each at a single and at a fold-fused reading, and the rows below are the readings this manuscript reports. Values carrying a standard deviation are means over the five per-fold models, and on those rows the detection recall and classification accuracy columns are the same five-model means printed without their dispersion. All rows except T1 use the axis-resolved label space L2.

Configuration	Reading	AP50	Detection Recall (%)	Classification Accuracy (%)	Macro-F1	Graded Correctly (%)
T1 binary	single	57.24 ± 3.15	92.4	68.94	68.44 ± 2.41	63.72
T1 binary	fold-fused	66.14	93.5	72.70	72.54	68.00
T2 baseline	single	45.23 ± 1.92	91.6	58.02	55.38 ± 2.34	53.16
T2 baseline	fold-fused	55.30	92.0	60.54	58.11	55.69
T3 at 1280 with TTA	single	51.06 ± 1.40	95.8	59.77	57.49 ± 1.98	57.23
T3 at 1280 with TTA	fold-fused	56.84	95.7	62.38	59.60	59.69
H7 wavelet 1280 with TTA	single	52.46 ± 1.59	96.0	62.05	59.73 ± 1.84	59.57
H7 wavelet 1280 with TTA	fold-fused	57.40	96.9	63.17	60.20	61.23
H2 wavelet 1024 with TTA	fold-fused	58.39	96.0	60.90	59.20	58.46
RT-DETR at 1280	single	52.89 ± 1.54	96.3	60.26	56.15 ± 2.22	58.04
Three-member ensemble	per fold	56.58 ± 1.66	96.0	62.05	59.68 ± 1.24	59.57
Three-member ensemble	full	59.09	96.3	62.94	60.99	60.62
Two-member ensemble	full	58.76	96.3	61.66	59.48	59.38

**Table 9:** Operating characteristics of the fold-fused binary decision on the locked test set, with Wilson 95% intervals. End-to-end sensitivity counts every defective reference filling the detector did not find as a false negative, so its denominator is all 169 defective fillings of the locked test set, 9 of which received no matched box.

Quantity	Value (%)	95% CI	Count
Sensitivity	76.25	69.09 to 82.18	122/160
Specificity	68.75	60.78 to 75.75	99/144
Positive predictive value	73.05	65.86 to 79.21	122/167
Negative predictive value	72.26	64.24 to 79.07	99/137
Accuracy	72.70	67.43 to 77.40	221/304
End-to-end sensitivity	72.19	65.00 to 78.39	122/169

**Table 10:** Comparison with published work on radiographic assessment of root canal filling quality and on detection of root-filled teeth, quality grading first and presence detection second. NR denotes not reported. The metrics in the final column differ between studies and are named in each cell, so the values are not comparable across rows.

Study	Modality	Task and Labels	Images/Teeth	Validation	Held-Out	Separated	CI	Reported Performance
Çelik et al. [20]	Periapical	Length only; 3 categories	597/1121	5-fold CV	NR	No	No	Segmentation Dice 88%
Ayhan et al. [17]	Periapical, ex vivo	Length only; 2 classes	735/1971	Single split	Yes, 80	No	No	mAP50 87.89%
Jin et al. [16]	Periapical	Binary; length and density merged	1508/NR	Single split	External cohort	No	Kappa only	F1 92.05%/82.93%
Kazimierczak et al. [19]	Panoramic	6 independent binary features	55/1330 (all teeth)	Commercial tool	Whole cohort	Partly	No	Density acc. 62.79%
Kazimierczak et al. [18]	Panoramic + CBCT	5 independent binary features	163 patients/189	Commercial tool	Whole cohort	Partly	95% CI	Density F1 42.67%
Choi et al. [11]	Panoramic	Presence only	1638/NR	Single split	Yes, 20%	n/a	No	AR 89.2% at IoU 0.5
Khurshid et al. [12]	Panoramic	Presence only	2235/11,462	Single split, 3 centres	Yes, 200	n/a	No	mAP50 82.0%, all classes
Kazimierczak et al. [38]	Panoramic	Presence only	147 patients/4148	Commercial tool	Whole cohort	n/a	95% CI	Sensitivity 88.6%
This study	Panoramic	Nested; L1 binary, L2 3 classes	806/1668 roots	5-fold CV	Yes, 160 locked	Yes	95% CI	Det. 92.0%/class. 60.54%

## Data Availability

The minimal dataset supporting the findings of this study, comprising the adjudicated annotations under pseudonymised image identifiers, the data partition, the model predictions, the measurement files, including the experiment ledger, and the analysis code, is openly available in Zenodo at https://doi.org/10.5281/zenodo.22956835 [53].

## Abbreviations

The following abbreviations are used in this manuscript:

AAO	Apical attribution overlap
AP	Average precision
AP50	Average precision at an intersection-over-union of 0.50
AP75	Average precision at an intersection-over-union of 0.75
AR	Average recall
BRF	Box-and-ring fraction of the attribution mass
CAR	Coronal-to-apical attribution ratio
CBCT	Cone beam computed tomography
CI	Confidence interval
COCO	Common Objects in Context
CV	Cross-validation
D-RISE	Detector randomised input sampling for explanation
F1	Harmonic mean of precision and recall
GFLOPs	Giga floating-point operations
IoU	Intersection over union
L1	Binary label space, adequate against defective
L2	Axis-resolved label space, adequate against length against density
mAP50	Mean average precision at an intersection over union of 0.50
NRT	Native-resolution texture encoding
RASA	Root-axis strip attention
RISE	Randomised input sampling for explanation
ROC	Receiver operating characteristic
RT-DETR	Real-time detection transformer
TTA	Test-time augmentation
VAO	Void attribution overlap
WVB	Wavelet void branch
